# Plant-Based Diet Indices with Greenhouse Gas Emissions and Risk of Cardiometabolic Diseases and All-Cause Mortality: Longitudinal China Cohort Study

**DOI:** 10.3390/nu17071152

**Published:** 2025-03-26

**Authors:** Yiqian Lv, Man Wu, Wenjing Liu, Ke Liu, Yin Wang, Zhixin Cui, Qishan Ma, Huicui Meng

**Affiliations:** 1 School of Public Health (Shenzhen), Shenzhen Campus of Sun Yat-Sen University, Sun Yat-Sen University, Shenzhen 518107, China; lvyq7@mail2.sysu.edu.cn (Y.L.); wuman5@mail2.sysu.edu.cn (M.W.); liuwj87@mail2.sysu.edu.cn (W.L.); liuk227@mail2.sysu.edu.cn (K.L.); wangy2287@mail2.sysu.edu.cn (Y.W.); cuizhx3@mail2.sysu.edu.cn (Z.C.); 2 Shenzhen Center for Disease Control and Prevention, Shenzhen 518055, China; 3 Guangdong Provincial Key Laboratory of Food, Nutrition and Health, Guangzhou 510080, China; 4 Guangdong Province Engineering Laboratory for Nutrition Translation, Guangzhou 510080, China

**Keywords:** plant-based diet index, longitudinal cohort study, cardiometabolic diseases, all-cause mortality, greenhouse gas emissions

## Abstract

**Background**: Environmental and cardiometabolic impacts of adherence to plant-based dietary patterns with different quality are unclear. **Objectives:** To investigate the associations between adherence to the overall, healthy, and unhealthy plant-based dietary patterns, as assessed by the plant-based diet index (PDI), healthy PDI (hPDI), and unhealthy PDI (uPDI), respectively, and risk of myocardial infarction (MI), type 2 diabetes (T2D), stroke, and all-cause mortality and greenhouse gas (GHG) emissions. **Methods**: Data from adults (N = 14,652 for cardiometabolic diseases and 15,318 for all-cause mortality) in the China Health and Nutrition Survey (1997–2015 wave) were analyzed. PDI, hPDI, and uPDI scores were calculated with dietary intake data. The total GHG emissions were calculated by summing the amount of emissions from all food groups included in the index. Cox proportional hazard regression models and linear regression models were used for statistical analysis. **Results**: Greater adherence to an unhealthy plant-based dietary pattern, as reflected by higher uPDI scores, was positively associated with risk of MI (Q5 vs. Q1: HR = 5.90; 95% CI: 2.59–13.48), T2D (Q5 vs. Q1: HR = 2.18; 95% CI: 1.75–2.73), stroke (Q5 vs. Q1: HR = 5.96; 95% CI: 2.86–12.42) and all-cause mortality (Q5 vs. Q1: HR = 6.87; 95% CI: 4.70–10.03). PDI scores were inversely associated with the risk of MI, T2D, and all-cause mortality, and hPDI scores were inversely and positively associated with the risk of T2D and stroke, respectively. All scores were inversely associated with GHG emissions (all *p*-trends < 0.001). **Conclusions**: Long-term adherence to unhealthy plant-based dietary patterns guided by higher uPDI scores may be a risk factor for new-onset cardiometabolic diseases and all-cause death in Chinese adults. Food-based dietary guidelines, clinicians, and dietitians should consider the quality of plant-based dietary patterns prior to making recommendations for both healthy individuals and those with elevated cardiometabolic disease risk.

## 1. Introduction

Cardiometabolic diseases (CMDs), including cardiovascular diseases, stroke, type 2 diabetes (T2D), and metabolic syndrome, contribute to the top ten leading causes of disability and premature adult death in China and globally, accounting for over 40% of all-cause mortality in China [1,2]. Modifiable risk factors, such as unhealthy diets, physical inactivity, and smoking, play critical roles in the development of CMDs [3]. Among these factors, diet is a key risk factor that not only influences the risk of CMDs and premature adult death but also influences environmental sustainability [4]. Based on the Global Burden of Disease database 1990–2019, adherence to unhealthy dietary patterns, especially lower consumption of healthy plant-based foods and higher consumption of unhealthy plant-based and animal-based foods, is a major risk factor of premature adult death and Disability-Adjusted Life Years attributable to CMDs [5,6]. Moreover, healthy dietary patterns and lifestyles also play critical roles in improving the resilience and health outcomes of vulnerable populations, such as T2D patients, especially during challenging times such as the COVID-19 pandemic [7].

The ecological footprint measures how much nature, expressed in the common unit of ‘bioproductive space with world average productivity’, is used exclusively for producing all the resources a given population consumes and absorbing the waste they produce [8]. Greenhouse gas (GHG) emissions are key contributors to the ecological footprint, as they drive climate change [9]. Among all food groups, animal-based foods, such as red and processed meat, are associated with a higher risk of CMDs, premature death, and higher emissions of GHG [10]. On the contrary, plant-based foods, such as whole grains, legumes, fruits, and vegetables are associated with lower risk of CMDs and premature death and lower emissions of GHG [10]. Several plant-based dietary patterns, characterized by reduced consumption of animal-derived foods and increased consumption of plant-derived foods, have been developed in recent years [4,11]. However, plant-based foods are not equally beneficial, and plant-based dietary patterns focusing on different types or quality of plant-based foods may have different impacts on population health and environmental sustainability [10]. Transitioning to sustainable diets, which are defined as diets that are healthy, nutrient-dense, accessible, affordable, culturally acceptable, and environmental-friendly, can collectively provide long-term benefits for both planetary and population health [12,13]. Evaluation of available metrics of plant-based dietary patterns to find a standardized or unified tool for the purpose of promoting sustainable diet, population, and planetary health is an urgent requirement. This information is essential in the food-based dietary guidelines for CMD prevention and sustainable public health policies for improving environmental health.

The plant-based diet index (PDI), healthy PDI (hPDI), and unhealthy PDI (uPDI) have been developed to assess adherence to overall, healthy, and unhealthy plant-based dietary patterns [11]. The PDI emphasizes plant-based foods and minimizes animal-based foods with higher scores representing higher consumption of plant-based foods, regardless of food quality [11]. The hPDI focuses on healthy plant-based foods and minimizes unhealthy plant-based foods and animal-based foods with higher scores representing a diet enriched in healthy and nutrient-dense plant-based foods [11]. The uPDI emphasizes unhealthy plant-based foods while minimizing animal-based foods, and higher scores of uPDI indicate a diet high in unhealthy and energy-dense plant-based foods, which may be linked to adverse health outcomes [11]. Although inverse associations between PDI and hPDI with risk of CMDs [11,14,15,16,17,18,19,20,21,22,23] and all-cause mortality [14,18,20,24,25,26,27] and positive associations between uPDI with risk of CMDs [11,15,16,20,28] and all-cause mortality [20,25,26,27,29] have been documented in several cross-sectional and prospective cohort studies, other studies have reported null associations [14,18,23,24,30,31,32,33,34,35,36,37]. While these indices have been validated in diverse populations, the majority of previous studies have primarily focused on Western populations. Available data on the associations between plant-based diet indices and risk of CMDs and all-cause mortality in the Chinese population are limited to only four cohort studies focusing on the PDI, hPDI, and uPDI with the risk of T2D or all-cause mortality [27,29,38,39], with no attention given to other CMDs, such as myocardial infarction (MI) and stroke. There was also no study on the relationship between the uPDI and the risk of CMDs in the Chinese population. In addition to population health, only one study explored the environmental impacts of adherence to plant-based dietary patterns, as assessed by the PDI, hPDI, and uPDI, in the Chinese population [29].

### Aims

The main objective of the current study was to investigate the associations between adherence to the overall, healthy, and unhealthy plant-based dietary patterns, evaluated by the PDI, hPDI, and uPDI, respectively, with the risk of new-onset CMDs and all-cause mortality in a nation-wide prospective cohort of Chinese adults. The secondary objectives were to explore the relationship between these dietary patterns and GHG emissions. We hypothesized that higher PDI and hPDI scores, which represented higher adherence to overall and healthy plant-based dietary patterns, were inversely associated with risk of new-onset CMDs and all-cause mortality as well as GHG emissions in Chinese adults, while the uPDI had positive associations.

## 2. Materials and Methods

### 2.1. Study Population

Data used in this study were obtained from the China Health and Nutrition Survey (CHNS), which is a longitudinal cohort study initiated in 1989 to track the health and nutritional changes of the Chinese population during rapid social and economic evolution.

From 1989 to 2015, CHNS completed ten waves of surveys in more than 7200 households and 30,000 participants from fifteen provinces with diverse geographic and economic characteristics. A detailed overview of the objective and design of CHNS has been provided in a prior study [40]. Research protocols of CHNS received approval from the Institutional Review Committees of the University of North Carolina at Chapel Hill and the National Institute for Nutrition and Health at the Chinese Center for Disease Control and Prevention [41,42]. All participants signed written informed consent prior to participating in the survey. This study has been reported in accordance with the STROBE checklist (Table S1).

Data from the CHNS 1997–2015 wave were analyzed to investigate the prospective associations between adherence to the overall, healthy and unhealthy plant-based dietary patterns, as evaluated by the PDI, hPDI and uPDI, respectively, with the risk of new-onset CMDs (cohort A), including MI, T2D, and stroke and all-cause mortality (cohort B) as well as GHG emissions.

A total of 33,314 participants were initially selected in the cohorts A and B. The baseline for each participant was defined as the date of their first dietary intake survey during the 1997–2015 wave. In both cohorts A and B, participants were excluded from the initial cohort if they were younger than 18 years old at baseline, had no records from the 3-day consecutive 24-h dietary recalls, had no records in all physical examination data, participated in only one wave of the survey, or had no dietary data collected from food weighing method. Additionally, we further excluded participants with an implausible cumulative average of total energy intake (<800 or >4200 kcal/day for men; <500 or >3500 kcal/day for women) [43] and those who were breastfeeding or pregnant. In cohort A, participants were also excluded if they were diagnosed with CMDs or tumors or took medicines to treat these diseases at baseline, and there was a total of 14,652 participants, including 7428 males and 7224 females free from CMDs or tumors at baseline in the final analysis (Figure S1A). The Cohort B included a total of 15,318 participants (7760 males and 7558 females) (Figure S1B).

### 2.2. Dietary Intake Assessment and Calculation of Indices for Plant-Based Dietary Patterns

Information on dietary intake assessment and calculations of food and nutrient intakes has been summarized in detail previously [40]. Briefly, dietary intake data were collected at the individual level through 3-day consecutive of 24-h dietary recalls under the guidance of trained interviewers, which was based on a balance between capturing dietary variability and minimizing participant burden. The intakes of cooking oils and condiments were measured at the household level through a food-weighing method [40]. The feasibility of these assessment methods has been validated in previous studies [40]. The method of calculation of food and nutrient intakes has been summarized in detail previously [40]. The intake of a specific food group was calculated by summing up the intakes of all foods within this food group. The daily intakes of food groups, foods, or nutrients were averaged over three days and were adjusted according to total energy intakes with the residual method [44]. Dietary records were updated during each survey wave for a total of six waves between 1997 and 2011. Cumulative average values of daily food group, food, and nutrient intakes from baseline to the date of diagnosis with CMDs, death, or the end of follow-up were calculated in the analysis to reduce intra-individual variations and reflect the long-term dietary habits of participants [44].

Adherence to the overall, healthy and unhealthy plant-based dietary patterns was assessed using the PDI, hPDI and uPDI, respectively. These indices were computed based on previously established methods developed by Satija et al. [11]. A total of seventeen food groups were involved in the computation, including seven healthy plant-based food groups (whole grains, fruits, vegetables, nuts, legumes, vegetable oils, and tea and coffee), five unhealthy plant-based food groups (fruit juices, refined grains, potatoes and starch, sugar-sweetened beverages, and sweets and desserts), and five animal-based food groups (animal fat, dairy, eggs, fish or seafood, and meat). Due to the extremely low intakes of omnivorous animal-based foods (including frogs, snakes, and scorpions) in Chinese adults, this food group was not included in the computation. For each food group, participants were categorized into quintiles (Q) based on the amounts of food group intakes. For specific food groups (including whole grains, fruits, nuts, tea and coffee, fruit juices, sugar-sweetened beverages, sweets and desserts, animal fat and dairy) that could only be categorized into quartiles, participants with no intake were allocated to the lowest quintile, while other participants were divided into quartiles.

In the computation of the PDI, each participant was assigned positive scores for consuming both healthy and unhealthy plant-based foods. Participants were assigned a score from one to five for plant-based food groups when their intakes fell in Q1 to Q5, respectively. Conversely, animal-based food groups were inversely scored, with Q1 receiving five points and Q5 receiving one point (Table S2). The final PDI score was calculated by summing the scores for all food groups, with a higher score indicating greater adherence to a plant-based diet.

In the computation of the hPDI, each participant was assigned positive scores for consuming healthy plant-based foods. Participants were assigned a score from one to five for healthy plant-based food groups when their intakes fell in Q1 to Q5, respectively. Conversely, unhealthy plant-based food groups and animal-based food groups were inversely scored, with Q1 receiving five points and Q5 receiving one point (Table S2). The final hPDI score was calculated by summing the scores for all food groups, with a higher score indicating greater adherence to a healthy plant-based diet.

In the computation of uPDI, each participant was assigned positive scores for consuming unhealthy plant-based foods. Participants were assigned a score from one to five for unhealthy plant-based food groups when their intakes fell in Q1 to Q5, respectively. Conversely, healthy plant-based foods and animal-based foods were inversely scored, with Q1 receiving five points and Q5 receiving one point (Table S2). The final uPDI score was calculated by summing the scores for all food groups, with a higher score indicating greater adherence to an unhealthy plant-based diet.

### 2.3. Assessment of GHG Emissions

In order to assess the environmental impacts of adherence to overall, healthy, and unhealthy plant-based dietary patterns, GHG emissions from the entire food production cycle including agricultural production, land use change, processing and packaging, transportation, retail, and consumption, were calculated using a previously established life cycle assessment (LCA) method [45]. The LCA method collected data on GHG emissions per gram of various types of foods from over 100 life cycle assessment studies. These studies considered GHG emissions throughout the entire life cycle process of each food type, from the initial production stage (such as raw material extraction) to the point when the final product leaves the farm gate [45]. The components of the food production cycle included crop production, livestock production, fisheries and aquaculture, and food waste [9]. Average GHG emissions per gram in the unit of gram CO_2_ equivalents (gCO_2e_) of each food group were estimated from the CHNS dataset [45] and multiplied by the amount of food group intakes of each participant to get the GHG emissions of each food group. The total GHG emissions related to each index were calculated by summing the amount of emissions from all seventeen food groups included in the index and expressed in units of gCO_2e_. Based on existing literature, GHG emissions from Chinese food systems were higher than global averages [46], partially due to higher nitrogen application [47] and lower nitrogen use efficiency [48], which may be attributed to several reasons, including high rice production, increasing meat consumption, as well as dietary shifts from plant-based patterns to animal-based patterns [9,49,50].

### 2.4. Ascertainment of CMDs and Death

The main outcomes of this study were the risk of new-onset CMDs, including MI, T2D, and stroke, and all-cause mortality between 2000 and 2015 in the CHNS cohort. Ascertainment of CMDs has been previously described in detail [40]. The death status of participants was confirmed by household members in each survey year. Data on cause-specific mortality were not available.

In case of conflicting records on health or death status in different waves, priority was given to the record from the first wave. The wave when participants had their first dietary intake survey during the 1997–2015 wave was used as the baseline wave. The follow-up time was the period from baseline to the initial diagnosis of MI, T2D, or stroke, or until death, or until the last wave before leaving the survey or the end of wave 2015, whichever occurred first. If the date of the dietary survey was not available, priority was given to the date of answering questionnaires from the same wave. Outcome time was calculated as the time of occurrence of the new-onset MI, T2D, stroke cases, or death cases during the follow-up period.

### 2.5. Assessment of Covariates

Information collection on sociodemographic and lifestyle characteristics and anthropometric measurements of participants were conducted using established methods as described previously [40]. Participants were geographically categorized into northern or southern China based on the Qinling–Huaihe line as the dividing boundary [51]. Educational levels were classified into three categories: primary (primary school or lower), middle (middle school), or high (high school or above). The urbanization level of communities was assessed using a composite index comprising 12 components, such as population density, economic activity, traditional and modern markets, transportation infrastructure, sanitation, communications, housing, education, diversity, and social services, as previously described [52], and the urbanization index was further divided into tertiles as low, moderate, or high. Smoking and drinking status were consistent with prior studies that explored the associations between dietary factors and risk of new-onset CMDs [53,54] or all-cause mortality [55,56] using the CHNS cohort. Physical activity levels were calculated based on the duration and intensity of occupational, household, leisure-time, and transportation activities, measured using a validated self-reported questionnaire, and expressed as metabolic equivalent-hour per week (MET-h/wk) [57].

### 2.6. Statistical Analysis

Statistical analysis was performed with SAS 9.4 for Windows (SAS Institute, Cary, NC, USA). Differences in baseline characteristics and daily intakes of food groups and nutrients among quintiles of PDI, hPDI, and uPDI scores were compared with the Kruskal–Wallis rank-sum analysis for continuous variables and the Chi-Square test for categorical variables. Participants were divided into five groups according to quintiles of PDI, hPDI, and uPDI scores. Cumulative average values of dietary intakes, body mass index (BMI), urbanization index, and physical activity data from baseline to the date of diagnosis with CMD outcomes, death, or the end of follow-up were used in the analysis to reduce intra-individual variations and represent long-term dietary habits [44]. In order to avoid confounding related to changes in dietary intakes or lifestyle behaviors of diagnosis with CMDs, data for cumulative average calculations were not included after diagnosis in the analysis with new-onset CMDs as primary outcomes. Cox proportional hazard regression models were used to assess the associations between PDI, hPDI, and uPDI scores and the risk of CMDs and all-cause mortality.

The independent variables were quintiles of PDI, hPDI, and uPDI scores, and the lowest quintile of each index was used as the reference group to estimate the hazard ratio (HR) and 95% confidence intervals (95%CI) for the risk of CMDs and all-cause mortality. All outcomes were fitted in separate Cox models. Model 1 adjusted for sex (male or female) and age (<50, 50–54, 55–59, 60–64, or ≥65 years). Model 2 additionally adjusted for BMI (underweight, normal weight, overweight or obese), region (northern or southern), urbanization index (tertiles), educational level (primary, middle or high), physical activity (tertiles), baseline hypertension [no (systolic blood pressure (SBP) < 140 mmHg and diastolic blood pressure (DBP) < 90 mmHg) or yes (SBP ≥ 140 mmHg or DBP ≥ 90 mmHg)], smoking status (yes or no), alcohol consumption (yes or no), and total energy intake (quintiles). Model 2 for stroke also adjusted for the dietary sodium: potassium ratio as an additional confounding factor. A test for linear trend was performed by using PDI, hPDI, and uPDI scores as continuous variables and assigning the median values of Q1–Q5 of these indices to the variables in the Cox regression model. Assessments of the proportional hazards assumption were conducted prior to performing Cox regression analysis. If certain independent variables and covariates did not meet the proportional hazards assumption, time-dependent variables constructed as time x variable interaction were incorporated into the Cox regression model.

Linear regression models were conducted to estimate the Least-Squares Means (95% confidence limit, 95% CL) of amounts of GHG emissions to investigate the associations between PDI, hPDI, and uPDI scores and the amount of GHG emissions. The same confounding factors were adjusted as models 1 and 2 of the Cox regression models.

Stratified analysis and potential effect modification were conducted for the associations between PDI, hPDI, and uPDI scores and risk of CMDs and all-cause mortality based on age (<60 or ≥60 years), sex (male or female), BMI (<24 or ≥24 kg/m^2^), region (northern or southern) and baseline hypertension (yes or no). Confounding factors were consistent with model 2 of the Cox regression model, and stratification variables were not adjusted in the corresponding models. The choice of age and BMI cut-off points in the CHNS analysis was based on the demographic and epidemiological context of the Chinese population, as well as regional health guidelines [54,58]. Several sensitivity analyses were conducted to avoid the potential impact of reverse causation. The associations were re-analyzed by excluding cases of CMDs or death that occurred during the first two years of follow-up, or via recomputed hPDI with positive coding for dairy products and fish and seafood, or via iteratively recomputed three indices by excluding each food group. Analysis with all-cause mortality was also conducted by excluding participants who had CMDs or tumors or took medicines to treat these diseases at baseline. A two-tailed *p* < 0.05 was considered statistically significant in all statistical analyses.

## 3. Results

### 3.1. Baseline Sociodemographic, Anthropometric, and Lifestyle Characteristics and Dietary Intakes of Study Participants

The prospective cohort study included 14,652 participants in cohort A with new-onset CMDs and 15,318 participants in cohort B with all-cause mortality as the primary outcome. A total of 280 (147,183 person-years) new-onset cases of MI, 1051 (143,849 person-years) new-onset cases of T2D, 404 (146,982 person-years) new-onset cases of stroke, and 1343 (154,485 person-years) cases of deaths were identified following a mean follow-up of ten years. Baseline characteristics of participants based on quintiles of PDI, hPDI, and uPDI scores are presented in Table 1, Table 2 and Tables S3–S5.

In cohort A, participants with higher PDI and uPDI scores were more likely to be male participants, have lower educational levels, live in low urbanized regions and northern China, smoke and drink alcohol, and be physically active at high intensity. In addition, they were more likely to have dietary intakes high in both healthy plant-based foods (whole grains) and unhealthy plant-based foods (refined grains and potatoes and starch), low in animal-based foods (dairy, eggs, fish or seafood, and meat), cholesterol and vitamin A and B_12_. Participants with higher hPDI scores were more likely to be female participants, live in highly urbanized regions, and be physically active at low intensity. Moreover, they were more likely to have dietary intakes high in whole grains, fruits, nuts, legumes, and polyunsaturated fatty acids (PUFAs), and low in animal-based foods and the majority of unhealthy plant-based foods and several nutrients (all *p* < 0.001) (Table 1, Table 2 and Table S4).

In cohort B, participants with higher PDI and uPDI scores were more likely to be male participants, live in low urbanized regions and northern China, smoke and be physically active at high intensity, and have dietary intakes high in unhealthy plant-based foods (potatoes and starch), low in animal-based foods (dairy, eggs, fish or seafood and meat), cholesterol and vitamin A and B_12_. Participants with higher hPDI scores were more likely to be female participants, live in highly urbanized regions and northern China, be physically active at low intensity, and have dietary intakes high in whole grains, fruits, legumes, and PUFAs, and low in animal-based foods, majority of unhealthy plant-based foods and several nutrients (all *p* < 0.001) (Tables S3 and S5).

### 3.2. Associations Between Adherence to Overall, Healthy, and Unhealthy Plant-Based Dietary Patterns with Risk of New-Onset CMDs and All-Cause Mortality

In fully adjusted models, higher PDI scores, which represented higher adherence to the overall plant-based dietary patterns, were associated with 52%, 66%, and 43% decreases in the risk of MI (model 2; Q5 vs. Q1: HR = 0.48; 95% CI: 0.28–0.85; *p*-trend = 0.031), T2D (model 2; Q5 vs. Q1: HR = 0.34; 95% CI: 0.26–0.46; *p*-trend < 0.001) and all-cause mortality (model 2; Q5 vs. Q1: HR = 0.57; 95% CI: 0.44–0.74; *p*-trend < 0.001) in comparison to lower PDI scores, respectively (Table 3). However, there was no significant association between PDI and the risk of stroke (Table 3).

Higher hPDI scores, which represented higher adherence to healthy plant-based dietary patterns, were associated with a 19% decrease in risk of T2D (model 2; Q5 vs. Q1: HR = 0.81; 95% CI: 0.65–0.99; *p*-trend = 0.039) in comparison to lower hPDI scores in the fully adjusted models (Table 3). Conversely, there were positive associations between hPDI and risk of stroke (model 2; Q5 vs. Q1: HR = 1.44; 95% CI: 1.00–2.09; *p*-trend = 0.038) (Table 3). There were no significant associations between hPDI and risk of MI and all-cause mortality (Table 3).

Higher uPDI scores, which represented higher adherence to unhealthy plant-based dietary pattern, were positively associated with 4.90, 1.18, 4.96 and 5.87 higher folds of risk of MI (model 2; Q5 vs. Q1: HR = 5.90; 95% CI: 2.59–13.48; *p*-trend < 0.001), T2D (model 2; Q5 vs. Q1: HR = 2.18; 95% CI: 1.75–2.73; *p*-trend < 0.001), stroke (model 2; Q5 vs. Q1: HR = 5.96; 95% CI: 2.86–12.42; *p*-trend < 0.001) and all-cause mortality (model 2; Q5 vs. Q1: HR = 6.87; 95% CI: 4.70–10.03; *p*-trend < 0.001) in comparison to lower uPDI scores, respectively, in the fully adjusted models (Table 3).

### 3.3. Associations Between Adherence to Overall, Healthy, and Unhealthy Plant-Based Dietary Patterns with Amounts of GHG Emissions

The Least-Squares Means of GHG emissions by quintiles of adherence to healthy and unhealthy plant-based dietary patterns in the study population are shown in Table 4. Regardless of outcomes, the amounts of GHG emissions decreased significantly across quintiles of PDI, hPDI, and uPDI scores in both models adjusted for sex and age and fully adjusted models (all *p*-trends < 0.001). Compared with participants in the lowest quintiles of PDI, hPDI and uPDI, participants in the highest quintiles had 34% to 57% lower amounts of GHG emissions (34%: Q5 of hPDI 3729.58 vs. Q1 of hPDI 5652.94 in model 2 of all-cause mortality; 57%: Q5 of PDI 2967.33 vs. Q1 of PDI 6869.88 in model 2 of all-cause mortality) (Table 4).

### 3.4. Associations Between Adherence to Overall, Healthy, and Unhealthy Plant-Based Dietary Patterns with Risk of New-Onset CMDs and All-Cause Mortality on the Basis of Potential Effect Modifiers

**Figure 1:**
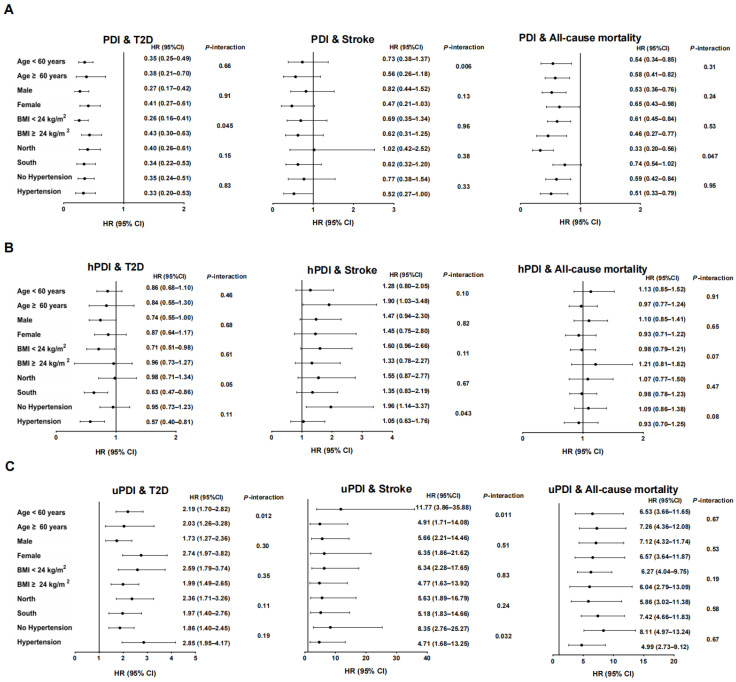
Associations between plant-based diet indices and risk of T2D, stroke (N = 14,652), and all-cause mortality (N = 15,318) in Chinese adults who participated in the China Health and Nutrition Survey 1997–2015 wave, stratified by age, sex, BMI, region and baseline hypertension history ((**A**): PDI and risk of T2D, stroke and all-cause mortality, (**B**): hPDI and risk of T2D, stroke and all-cause mortality, (**C**): uPDI and risk of T2D, stroke and all-cause mortality). Data were presented as HR (95% CI) of Q5 vs. Q1 estimated by Cox proportional hazard regression models. Models adjusted for sex, age, BMI, region, urbanization index, educational level, physical activity, baseline hypertension, smoking status, alcohol intake, and total energy intake. Models for stroke also adjusted for the sodium: potassium ratio. Stratification variables were not adjusted as confounding factors in the corresponding models. Abbreviations: BMI, body mass index; CI, confidence interval; hPDI, healthy PDI; HR, hazard ratio; PDI, plant-based diet index; Q, quintiles; T2D, type 2 diabetes; uPDI, unhealthy PDI. The corresponding numerical data are listed in Table S6.

There were significant effect modifications of the associations between PDI with T2D by BMI (*p*-interaction = 0.045), with stroke by age (*p*-interaction = 0.006), and with all-cause mortality by region (*p*-interaction = 0.047), respectively (Figure 1A, Table S6). PDI was associated with a greater reduced risk of T2D in underweight and normal-weight participants (Q5 vs. Q1: HR = 0.26; 95% CI: 0.16–0.41; *p*-trend < 0.001) than in overweight and obese participants (Q5 vs. Q1: HR = 0.43; 95% CI: 0.30–0.63; *p*-trend < 0.001) (Figure 1A, Table S6). There was an inverse association between PDI and risk of all-cause mortality in participants resident in northern China (Q5 vs. Q1: HR = 0.33; 95% CI: 0.20–0.56; *p*-trend < 0.001) rather than in southern China (Q5 vs. Q1: HR = 0.74; 95% CI: 0.54–1.02; *p*-trend = 0.09) (Figure 1A, Table S6).

There were significant effect modifications of the associations between hPDI with stroke by baseline hypertension (*p*-interaction = 0.043) (Figure 1B, Table S6). There was a positive association between hPDI and risk of stroke in participants without hypertension at baseline (Q5 vs. Q1: HR = 1.96; 95% CI: 1.14–3.37; *p*-trend = 0.020) rather than participants with hypertension at baseline (Q5 vs. Q1: HR = 1.05; 95% CI: 0.63–1.76; *p*-trend = 0.58) (Figure 1B, Table S6).

There were significant effect modifications of the associations between uPDI and T2D by age (*p*-interaction = 0.012) and uPDI and stroke by age (*p*-interaction = 0.011) and baseline hypertension (*p*-interaction = 0.032), respectively (Figure 1C, Table S6). The positive association between uPDI and risk of T2D was stronger in younger (Q5 vs. Q1: HR = 2.19; 95% CI: 1.70–2.82; *p*-trend < 0.001) than older (Q5 vs. Q1: HR = 2.03; 95% CI: 1.26–3.28; *p*-trend = 0.012) participants (Figure 1C, Table S6). Similarly, the positive association between uPDI and risk of stroke was also stronger in younger (Q5 vs. Q1: HR = 11.77; 95% CI: 3.86–35.88; *p*-trend < 0.001) than older (Q5 vs. Q1: HR = 4.91; 95% CI: 1.71–14.08; *p*-trend = 0.004) participants (Figure 1C, Table S6). The positive association between uPDI and risk of stroke was stronger in participants without hypertension at baseline (Q5 vs. Q1: HR = 8.35; 95% CI: 2.76–25.27; *p*-trend < 0.001) than those with hypertension at baseline (Q5 vs. Q1: HR = 4.71; 95% CI: 1.68–13.25; *p*-trend = 0.002) (Figure 1C, Table S6).

There was no evidence of effect modifications of the associations between PDI, hPDI, and uPDI with risk of MI (Figure S2, Table S6), between hPDI with risk of T2D, and between hPDI and uPDI with risk of all-cause mortality by age, sex, BMI, region or baseline hypertension (all *p*-interactions > 0.05) (Figure 1, Table S6).

The sensitivity analyses excluding cases of CMDs and deaths within two years of follow-up demonstrated similar results to the main analyses (Table 5). In the sensitivity analysis recomputing hPDI with positive coding for dairy products and fish and seafood, the re-coded hPDI scores were inversely associated with the risk of MI, T2D, and all-cause mortality, but were not significantly associated with risk of stroke (Table S7). In the sensitivity analysis with iteratively recomputing the PDI, hPDI, and uPDI scores by excluding each food group, similar results to the main analyses were observed for the associations between iteratively recomputed PDI with risk of T2D and all-cause mortality as well as uPDI with all outcomes (Table S8). The sensitivity analyses for the associations between PDI, hPDI, and uPDI with all-cause mortality, excluding participants with CMDs and cancer at baseline, also demonstrated similar results to the main analyses (Table 6).

## 4. Discussion

In the current study, higher PDI scores were associated with a reduced risk of MI, T2D, and all-cause mortality, and higher hPDI scores were associated with a reduced risk of T2D. These findings corroborated our hypothesis and were consistent with several studies reported previously [11,15,16,18,19,21,23,33,35,38,39]. Participants with higher PDI and hPDI scores consumed more whole grains, fruits, vegetables, nuts, legumes, and vegetable oils than those with lower scores. These food groups have been reported to relate to reduced risk of CMDs [59,60,61] and all-cause mortality [61,62], which may be attributed to their favorable effects on body weight management [63] and improving cardiometabolic risk factors, such as lipid and lipoprotein profiles [63,64], insulin sensitivity [65], and anti-oxidative capacity [64,66]. Healthy plant-based foods are enriched in dietary fiber, PUFAs, or polyphenolic compounds. These nutrients and bioactive compounds play an important role in anti-inflammation [67,68,69], maintaining gut microbiota homeostasis [10,70,71] and producing gut-derived bioactive metabolites [71,72], such as short-chain fatty acids, which may also contribute to the beneficial effects of dietary patterns with higher PDI and hPDI scores against CMDs and premature death. A previous study with the Chinese population has reported an inverse association between long-term PDI with relative abundance of gut *Peptostreptococcus*, a microbe positively associated with high-sensitivity C-reactive protein and inversely associated with high-density lipoprotein cholesterol. This study has suggested that long-term adherence to plant-based diets may be associated with alterations in gut microbial taxa, which may influence the risk of CMDs and all-cause mortality via modulating cardiometabolic risk factors in Chinese adults [70]. In addition, a prior study by our group, which is particularly relevant to Chinese adults, has demonstrated that higher PDI and hPDI scores are associated with lower inflammatory potential of diet [40], further suggesting the anti-inflammatory effects of dietary patterns with higher PDI and hPDI scores as potential mechanisms underlying the inverse associations between PDI and hPDI with risk of MI, T2D or all-cause mortality.

There was no significant association between PDI and the risk of stroke, and this finding was in line with previous studies [30,36]. However, higher hPDI scores were positively associated with an increased risk of stroke. This finding did not support our hypothesis. Prior studies have reported associations between vegetarians or very low intakes of animal-based foods with a higher risk of stroke [73]. In the current prospective cohort, when hPDI was recomputed by assigning positive scores to two animal-based food groups, including dairy products and fish or seafood, the recomputed hPDI scores were not associated with the risk of stroke. These findings collectively suggested the potential protective effects of animal-based foods on stroke prevention and may partially explain the positive associations between hPDI and the risk of stroke.

In the current study, higher uPDI scores were associated with increased risk of MI, T2D, stroke, and all-cause mortality, supporting our hypothesis. These findings were consistent with prior reports [11,15,16,20,25,26,27,28,29]. The uPDI has primarily focused on unhealthy plant-based food groups, such as refined grains, and potatoes and starch. Previously studies have reported the positive associations between higher intakes of these unhealthy plant-based food groups and increased risk of CMDs [59,60,74] and all-cause mortality [75]. Potential underlying mechanisms may be partially attributed to the lack of dietary fiber and higher energy contents, glycemic index, and glycemic load of these food groups, which contribute to cardiometabolic risk factors such as insulin resistance and dyslipidemia [76,77]. Furthermore, uPDI scores were positively associated with the high pro-inflammatory potential of diet [40], which has also been reported to contribute to increased risk of CMDs and all-cause mortality [40]. In the analysis with recomputed uPDI by excluding individual food groups, positive associations between recomputed hPDI and risk of CMDs and all-cause mortality remained unchanged. Conversely, the recomputed PDI and hPDI demonstrated sensitivity to the exclusion of specific food groups. These findings suggested uPDI as the most appropriate and precise index for assessing adherence to unhealthy plant-based dietary patterns and related health outcomes.

Age significantly modified the associations between uPDI and the risk of T2D and stroke. Compared with older adults, young and middle-aged adults demonstrated stronger positive associations between uPDI and risk of T2D and stroke. This finding was in agreement with a prior study in Chinese populations of the Chinese Longitudinal Healthy Longevity Study [27], yet the reasons were not obvious. Older adults are more adaptive to healthy plant-based dietary patterns [21]. Due to changes in metabolism and nutritional needs as well as concerns about overall diet quality, older adults may have lower adherence to unhealthy plant-based dietary patterns. BMI significantly modified the associations between PDI and risk of T2D, and the inverse association was stronger among participants with under or normal weights than overweight or obese participants, aligning with previous studies [11,35]. Additionally, the French prospective cohort study has reported BMI as a partial contributor to the potential impacts of plant-based dietary patterns on the development of T2D [35]. Excessive adiposity induced by overweight and obesity relates to several risk factors, including insulin resistance, systemic chronic low-grade inflammation, and increased secretion of free fatty acids [78,79], collectively contributing to the risk of T2D [80]. Adherence to plant-based dietary patterns may prevent T2D by improving these risk factors. Region significantly modified the associations between PDI with risk of all-cause mortality. Possible underlying causes of the region-specific differences may include regional disparities in prevalence of overweight and obesity, eating habits and behaviors as well as other lifestyle factors [81,82]. Although these factors were adjusted as potential confounders in the analysis. Baseline hypertension significantly modified the associations between hPDI and uPDI with the risk of stroke. Positive associations between hPDI and risk of stroke were only observed in participants without hypertension at baseline, and positive associations between uPDI and risk of stroke were stronger in participants without hypertension compared to those with hypertension at baseline. These findings suggested that the quality of plant-based dietary patterns has stronger impacts on healthy rather than hypertensive individuals. As hypertension is a risk factor for stroke, hypertensive individuals may follow relatively healthier lifestyles and dietary patterns and may counterbalance the impact of plant-based dietary patterns in these participants.

In the current study, higher PDI, hPDI, and uPDI scores were associated with reduced amounts of GHG emissions. The inverse associations between adherence to overall and healthy plant-based dietary patterns and amounts of GHG emissions supported our hypothesis and were in agreement with findings from the Nurses’ Health Study [83]. Of note, uPDI scores were also inversely associated with GHG emissions, which did not support our hypothesis. Based on a global modeling study [84], dietary patterns with low animal-based foods, such as vegan diet and flexitarian diet, could reduce GHG emissions by 54-87%. By design, PDI, hPDI, and uPDI focus on the quality of plant-based food groups and assign negative scores to all animal-based foods, which may partially explain the inverse associations between these indices and GHG emissions. Recently, another study has investigated the association between these indices with GHG emissions and all-cause mortality in middle-aged and older adults in China [29]. Their findings were consistent with the current study and have reported that higher uPDI scores are associated with a higher risk of all-cause mortality and lower GHG emissions. Lower GHG emissions contribute to climate change towards lower atmospheric concentrations of CO_2_, CH_4_, and N_2_O, aligning with global climate goals such as the Paris Agreement [50], which underscore the importance of dietary changes as a key strategy for achieving sustainable development goals and fostering a healthier planet [85].

In addition to the strengths summarized in a prior study [40], there are several other strengths of the current study. Adherence to the overall, healthy and unhealthy plant-based dietary patterns was assessed using PDI, hPDI, and uPDI, respectively, enabling comparisons and validations of these indices in Chinese adults. These indices utilized progressive scoring systems and evaluated the gradual increases in quantities and qualities of plant-based food groups without excluding animal-based food groups, which were more acceptable than dietary patterns with no or limited animal-based food groups. The study explored the co-benefits of adherence to plant-based dietary patterns by investigating its relationships with cardiometabolic outcomes and GHG emissions, partially overcoming the lack of environmental aspects in the initial development of PDI, hPDI, and uPDI. In addition, the study conducted multiple sensitivity analyses, which improved the reliability of the results.

## 5. Limitations

Our study has several limitations. Dietary intake data were self-reported, which may introduce recall bias. The use of 24 h dietary recall records, rather than food-frequency questionnaires, may not accurately reflect long-term dietary intakes. However, we employed cumulative average dietary intake data as exposure values to better represent long-term dietary habits. The indices for plant-based dietary patterns were developed according to the health effects of plant-based food groups reported in previous studies. The same weights were given to each food group in the computation of different indices. Adjustments in weights of food groups and scoring criteria may be required for individuals with different cultural and nutritional backgrounds considering the different impacts of food groups on health outcomes across diverse populations [11]. Unlike the indices for dietary patterns focusing on overall diet quality, such as the Healthy Eating Index and Mediterranean Diet Score, indices for plant-based dietary patterns pay more attention to the quality of plant-based food groups. The computation of PDI, hPDI, and uPDI assigns negative scores to all animal-based foods, which may undermine the potential beneficial effects of moderate intakes of certain animal-based foods on health outcomes. The data on the amount of GHG emissions per gram of different types of foods were collected from a previous study using the CHNS dataset and were not measured on our own. The identification of new-onset T2D cases was based on self-reported questionnaires rather than physician confirmation, which was impractical given the large scale of our cohort study. Nevertheless, this limitation was partially addressed by using fasting glucose and HbA1c data from the 2009 wave to confirm the occurrence of new-onset T2D cases. Data on cause-specific mortality was only available in 1991; hence, analysis was solely conducted with all-cause mortality in this study.

Additionally, our study was subject to the common limitations of observational research. Although we adjusted for potential confounders in our models, we could not entirely rule out the presence of other residual confounders. The results were derived from a single prospective cohort study, which may limit the generalizability of the findings to broader populations or different geographic and cultural contexts. In addition, causal relationships between the PDI, hPDI, and uPDI and the risk of CMDs and all-cause mortality could not be established in this prospective cohort, and the underlying mechanisms were not explored.

## 6. Conclusions

In conclusion, this study demonstrated long-term positive associations between adherence to unhealthy plant-based dietary patterns, as represented by higher uPDI scores, with increased risk of MI, T2D, stroke, and all-cause mortality in Chinese adults. Higher PDI scores were associated with a reduced risk of MI, T2D, and all-cause mortality, and higher hPDI scores were associated with a reduced risk of T2D and an increased risk of stroke. These findings indicated uPDI as a suitable and precise index for assessing adherence to unhealthy plant-based dietary patterns and its associations with health outcomes. Long-term adherence to unhealthy plant-based dietary patterns guided by a higher uPDI score may contribute to higher risk and burden of CMDs and all-cause death in Chinese adults, yet investigations of causal relationships through randomized controlled trials are necessary. Food-based dietary guidelines, clinicians and dietitians should consider the quality of plant-based dietary patterns prior to making recommendations for both healthy individuals and those with elevated CMD risk. Higher PDI, hPDI, and uPDI scores were associated with lower GHG emissions, and the impacts of these indices on environmental sustainability require further investigations with other environmental outcomes.

## Figures and Tables

**Table 1:** Baseline sociodemographic, anthropometric, and lifestyle characteristics of 14,652 Chinese adults in cohort A who participated in the China Health and Nutrition Survey 1997–2015 wave based on quintiles of plant-based diet indices *^a^*.

Variables	All	Quintiles of PDI			*p ^b^*	Quintiles of hPDI			*p ^b^*	Quintiles of uPDI			*p ^b^*
		Q1	Q3	Q5		Q1	Q3	Q5		Q1	Q3	Q5	
N	14,652	2381	2890	2986		2678	3281	3629		2882	2838	3513	
PDI	47 (43, 52)	39 (37, 40)	47 (46, 48)	56 (54, 58)	<0.001	49 (44, 54)	45 (41, 51)	48 (46, 51)	<0.001	45 (41, 49)	47 (43, 51)	50 (47, 55)	<0.001
hPDI	53 (48, 57)	51 (48, 54)	56 (50, 59)	50 (46, 55)	<0.001	43 (41, 45)	53 (52, 54)	60 (59, 62)	<0.001	56 (52, 60)	53 (48, 57)	50 (45, 54)	<0.001
uPDI	52 (48, 56)	49 (45, 53)	52 (48, 56)	51 (55, 59)	<0.001	55 (52, 59)	52 (48, 56)	50 (46, 53)	<0.001	43 (41, 45)	52 (51, 53)	59 (58, 62)	<0.001
Age, years	45 ± 15	46 ± 16	45 ± 15	43 ± 14	<0.001	41 ± 13	45 ± 15	48 ± 16	<0.001	48 ± 15	45 ± 15	42 ± 14	<0.001
Female, N (%)	7224 (49.3)	1295 (54.4)	1536 (53.1)	1081 (36.2)	<0.001	811 (30.3)	1591 (48.5)	2309 (63.6)	<0.001	1741 (60.4)	1354 (47.7)	1473 (41.9)	<0.001
BMI, kg/m^2^	22.5 (20.5, 25.0)	22.5 (20.4, 24.8)	22.6 (20.5, 25.0)	22.5 (20.6, 25.0)	0.45	22.1 (20.3, 24.5)	22.5 (20.6, 24.9)	22.9 (20.7, 25.4)	<0.001	23.4 (21.2, 25.6)	22.4 (20.5, 24.9)	21.8 (20.1, 24.0)	<0.001
SBP, mmHg	120.0 (110.0, 130.0)	120.0 (110.0, 130.0)	120.0 (110.0, 130.0)	120.0 (110.0, 130.0)	0.31	118.7 (109.3, 126.7)	120.0 (110.0, 130.0)	120.0 (110.0, 130.7)	<0.001	120.0 (110.0, 130.7)	120.0 (110.0, 130.0)	119.3 (110.0, 126.7)	<0.001
DBP, mmHg	78.7 (70.0, 83.3)	78.3 (70.0, 83.0)	78.7 (70.0, 83.3)	80.0 (70.0, 84.9)	0.21	78.0 (70.0, 83.3)	78.7 (70.0, 83.0)	79.3 (70.0, 84.7)	0.001	79.3 (70.7, 83.3)	78.7 (70.0, 83.3)	78.3 (70.0, 82.0)	<0.001
Education level, N (%)					<0.001				<0.001				<0.001
Primary	6883 (47.0)	993 (41.7)	1365 (47.2)	1455 (48.7)		1203 (44.9)	1490 (45.4)	1814 (50.0)		804 (27.9)	1332 (46.9)	2105 (59.9)	
Middle	4166 (28.4)	640 (26.9)	817 (28.3)	949 (31.8)		867 (32.4)	944 (28.8)	960 (26.4)		800 (27.8)	820 (28.9)	997 (28.4)	
High	3603 (24.6)	748 (31.4)	708 (24.5)	582 (19.5)		6088 (22.7)	847 (25.8)	855 (23.6)		1278 (44.3)	686 (24.2)	411 (11.7)	
Urbanization index, N (%)					<0.001				<0.001				<0.001
Low	4859 (33.2)	373 (15.7)	943 (32.6)	1468 (49.2)		1065 (39.8)	1061 (32.3)	1108 (30.5)		175 (6.1)	883 (31.1)	2207 (62.8)	
Medium	4899 (33.4)	997 (41.9)	1006 (34.8)	810 (27.1)		978 (36.5)	1088 (33.2)	1135 (31.3)		679 (23.5)	1172 (41.3)	960 (27.4)	
High	4894 (33.4)	1011 (42.5)	941 (32.6)	708 (23.7)		635 (23.7)	1132 (34.5)	1386 (38.2)		2028 (70.4)	783 (27.6)	346 (9.8)	
Region, N (%)					<0.001				<0.001				<0.001
Southern	8551 (58.4)	1801 (75.6)	1676 (58.0)	1304 (43.7)		1810 (67.6)	1969 (60.0)	1813 (50.0)		1828 (63.4)	1740 (61.3)	1735 (49.4)	
Northern	6101 (41.6)	580 (24.4)	1214 (42.0)	1682 (56.3)		868 (32.4)	1312 (40.0)	1816 (50.0)		1054 (36.6)	1098 (38.7)	1778 (50.6)	
Currently smoking, N (%)	4648 (31.7)	665 (27.9)	843 (29.2)	1208 (40.5)	<0.001	1180 (44.1)	1052 (32.1)	825 (22.7)	<0.001	644 (22.3)	919 (32.4)	1354 (38.5)	<0.001
Currently drinking alcohol, N (%)	5401 (36.9)	777 (32.6)	971 (33.6)	1371 (45.9)	<0.001	1287 (48.1)	1235 (37.6)	1004 (27.7)	<0.001	971 (33.7)	1058 (37.3)	1391 (39.6)	<0.001
Physical activity status, N (%)					<0.001				<0.001				<0.001
Low	4676 (31.9)	871 (36.6)	935 (32.4)	735 (24.6)		639 (23.9)	1025 (31.2)	1489 (41.0)		1220 (42.3)	893 (31.5)	719 (20.5)	
Medium	5232 (35.7)	1020 (42.8)	1052 (36.4)	914 (30.6)		872 (32.5)	1210 (36.9)	1269 (35.0)		1279 (44.4)	1078 (38.0)	889 (25.3)	
High	4744 (32.4)	490 (20.6)	903 (31.2)	1337 (44.8)		1167 (43.6)	1046 (31.9)	872 (24.0)		383 (13.3)	867 (30.5)	1905 (54.2)	

*^a^* Continuous variables were presented as mean ± SD or median (P25, P75), and the categorical variables were presented as N (%). *^b^* Kruskal–Wallis rank-sum analysis was used in continuous variables and Chi-Square test was used in the categorical variables to test significant differences across different quintiles of plant-based diet indices. Abbreviations: BMI, body mass index; DBP, diastolic blood pressure; hPDI, healthy PDI; PDI, plant-based diet index; Q, quintiles; SBP, systolic blood pressure; SD, standard deviation; uPDI, unhealthy PDI.

**Table 2:** Baseline daily intakes of food groups of 14,652 Chinese adults in cohort A who participated in the China Health and Nutrition Survey 1997–2015 wave based on quintiles of plant-based diet indices *^a^*.

Variables	All	Quintiles of PDI			*p ^b^*	Quintiles of hPDI			*p ^b^*	Quintiles of uPDI			*p ^b^*
		Q1	Q3	Q5		Q1	Q3	Q5		Q1	Q3	Q5	
Grains, g	425.3 ± 165.7	329.5 ± 103.1	403.2 ± 132.7	570.6 ± 193.5	<0.001	556.3 ± 181.3	420.4 ± 141.3	340.7 ± 122.6	<0.001	304.6 ± 101.3	411.2 ± 135.7	562.2 ± 166.8	<0.001
Whole grains, g	19.6 ± 57.7	4.5 ± 25.2	14.8 ± 41.0	47.0 ± 97.6	<0.001	9.7 ± 42.7	20.1 ± 64.1	26.1 ± 52.9	<0.001	15.9 ± 38.4	17.2 ± 47.0	24.0 ± 70.1	<0.001
Fruits, g	26.5 ± 70.6	17.8 ± 58.8	26.1 ± 70.7	33.9 ± 83.0	<0.001	14.7 ± 54.2	24.5 ± 70.1	38.8 ± 80.2	<0.001	69.5 ± 95.5	18.4 ± 67.2	5.1 ± 29.4	<0.001
Vegetables, g	272.3 ± 149.4	218.9 ± 114.0	263.5 ± 141.0	334.0 ± 171.6	<0.001	294.1 ± 155.8	262.7 ± 149.8	270.4 ± 140.9	<0.001	277.8 ± 134.9	274.0 ± 148.8	265.2 ± 163.2	<0.001
Nuts, g	3.2 ± 12.3	1.5 ± 7.7	2.7 ± 10.4	5.7 ± 17.7	<0.001	1.9 ± 12.1	3.4 ± 11.9	3.9 ± 12.3	<0.001	6.6 ± 15.3	2.7 ± 11.3	0.9 ± 7.7	<0.001
Legumes, g	49.0 ± 67.5	29.1 ± 46.8	45.5 ± 60.7	75.9 ± 91.0	<0.001	49.8 ± 69.7	47.3 ± 70.1	52.7 ± 66.2	<0.001	61.7 ± 68.7	49.8 ± 68.8	34.4 ± 63.2	<0.001
Vegetable oils, g	31.9 ± 29.1	21.5 ± 20.9	28.8 ± 25.9	45.7 ± 35.8	<0.001	29.7 ± 36.2	32.6 ± 28.7	31.7 ± 21.3	<0.001	34.8 ± 24.3	33.2 ± 33.2	26.1 ± 26.4	<0.001
Tea and coffee, g	1.3 ± 25.0	0.2 ± 9.7	1.4 ± 29.3	2.3 ± 35.2	<0.001	0.3 ± 7.6	1.1 ± 22.6	1.7 ± 29.7	0.007	4.6 ± 45.6	0.6 ± 15.5	0 ± 2.5	<0.001
Fruit juices, g	0.2 ± 5.6	0 ± 0.8	0.1 ± 2.3	0.1 ± 2.9	0.50	0.2 ± 6.6	0.2 ± 5.3	0 ± 0	0.033	0.3 ± 5.9	0.2 ± 8.4	0.1 ± 5.6	0.06
Refined grains, g	405.7 ± 158.9	325.0 ± 103.2	388.4 ± 134.9	523.6 ± 190.1	<0.001	546.7 ± 175.2	400.3 ± 127.3	314.7 ± 116.8	<0.001	288.7 ± 95.4	394.0 ± 129.4	538.2 ± 162.8	<0.001
Potatoes and starch, g	33.6 ± 63.3	11.8 ± 29.1	27.4 ± 51.2	67.3 ± 93.0	<0.001	54.8 ± 88.3	30.8 ± 56.6	21.9 ± 47.0	<0.001	15.6 ± 30.8	25.1 ± 45.2	65.7 ± 94.2	<0.001
Sugar-sweetened beverages, g	1.6 ± 19.3	0.4 ± 10.1	2.2 ± 26.1	2.5 ± 22.2	<0.001	2.6 ± 27.6	1.1 ± 12.2	0.3 ± 7.1	<0.001	2.6 ± 24.7	2.2 ± 23.2	0.6 ± 11.1	<0.001
Sweets and desserts, g	0.8 ± 7.1	0.3 ± 4.1	0.6 ± 5.6	1.7 ± 11.8	<0.001	1.5 ± 11.7	0.9 ± 7.0	0.3 ± 3.1	<0.001	0.9 ± 5.4	1.0 ± 7.9	0.4 ± 3.9	<0.001
Animal fat, g	6.8 ± 18.7	11.7 ± 21.2	6.6 ± 18.7	3.6 ± 12.6	<0.001	20.5 ± 32.7	4.2 ± 12.0	1.7 ± 7.4	<0.001	3.6 ± 12.1	9.2 ± 21.9	6.1 ± 17.8	<0.001
Dairy, g	14.8 ± 53.7	33.7 ± 77.5	10.9 ± 48.1	8.2 ± 41.8	<0.001	12.7 ± 50.5	19.4 ± 59.7	8.5 ± 40.9	<0.001	54.1 ± 91.7	5.2 ± 32.4	0.6 ± 11.7	<0.001
Eggs, g	23.2 ± 31.4	33.6 ± 35.3	20.9 ± 28.9	18.4 ± 29.8	<0.001	28.1 ± 36.2	25.4 ± 30.8	16.7 ± 26.4	<0.001	36.8 ± 32.1	23.2 ± 31.1	10.9 ± 23.4	<0.001
Fish or seafood, g	19.1 ± 34.2	34.6 ± 38.9	16.5 ± 30.7	11.0 ± 30.0	<0.001	25.4 ± 42.4	21.6 ± 33.0	10.3 ± 25.1	<0.001	36.0 ± 40.0	17.7 ± 31.9	6.3 ± 19.7	<0.001
Meat, g	76.5 ± 77.0	108.9 ± 68.2	75.4 ± 78.3	50.8 ± 73.0	<0.001	112.3 ± 100.7	75.5 ± 69.6	49.6 ± 55.0	<0.001	103.2 ± 69.9	85.6 ± 77.3	35.3 ± 55.6	<0.001

*^a^* Continuous variables were presented as mean ± SD. *^b^* Kruskal–Wallis rank-sum analysis was used to test significant differences across different quintiles of plant-based diet indices. Abbreviations: hPDI, healthy PDI; PDI, plant-based diet index; Q, quintiles; SD, standard deviation; uPDI, unhealthy PDI.

**Table 3:** Associations between plant-based diet indices and risk of MI, T2D, stroke (N = 14,652), and all-cause mortality (N = 15,318) in Chinese adults who participated in the China Health and Nutrition Survey 1997–2015 wave *^a^*.

Variables	Quintiles					*p*-Trend
	Q1	Q2	Q3	Q4	Q5	
**PDI**						
** MI**						
Median (range)	37 (23, 40)	43 (41, 45)	48 (46, 50)	53 (51, 55)	59 (56, 72)	
Cases (rate, %)	48 (1.75)	46 (1.61)	69 (2.24)	58 (2.13)	59 (1.82)	
Person year	28,559	31,043	33,075	26,868	27,638	
Model 1	1.00 (Ref)	0.78 (0.52–1.16)	1.01 (0.70–1.46)	0.90 (0.61–1.33)	0.85 (0.57–1.27)	0.69
Model 2	1.00 (Ref)	0.71 (0.47–1.07)	0.83 (0.56–1.25)	0.67 (0.42–1.08)	0.48 (0.28–0.85)	0.031
** T2D**						
Median (range)	37 (23, 40)	43 (41, 45)	48 (46, 50)	53 (51, 55)	59 (56, 72)	
Cases (rate, %)	225 (8.15)	206 (7.23)	223 (7.21)	190 (7.00)	207 (6.40)	
Person year	28,060	30,331	32,482	26,214	26,761	
Model 1	1.00 (Ref)	0.79 (0.65–0.95)	0.75 (0.62–0.90)	0.74 (0.61–0.90)	0.79 (0.65–0.97)	0.020
Model 2	1.00 (Ref)	0.71 (0.58–0.86)	0.57 (0.46–0.70)	0.46 (0.36–0.58)	0.34 (0.26–0.46)	<0.001
** Stroke**						
Median (range)	37 (23, 40)	43 (41, 45)	48 (46, 50)	53 (51, 55)	59 (56, 72)	
Cases (rate, %)	60 (2.20)	75 (2.62)	99 (3.21)	81 (2.97)	89 (2.74)	
Person year	28,391	31,175	33,109	26,693	27,614	
Model 1	1.00 (Ref)	1.02 (0.73–1.44)	1.23 (0.89–1.70)	1.18 (0.84–1.67)	1.25 (0.89–1.76)	0.14
Model 2	1.00 (Ref)	0.91 (0.65–1.29)	0.94 (0.66–1.33)	0.78 (0.52–1.17)	0.65 (0.40–1.04)	0.08
** All-cause mortality**						
Median (range)	38 (25, 41)	44 (42, 45)	48 (46, 50)	53 (51, 56)	60 (57, 75)	
Cases (rate, %)	213 (7.27)	216 (7.99)	288 (8.48)	319 (9.89)	307 (10.01)	
Person year	30,524	29,734	37,540	31,833	24,854	
Model 1	1.00 (Ref)	0.97 (0.80–1.17)	0.89 (0.75–1.06)	0.95 (0.80–1.14)	1.00 (0.83–1.20)	0.91
Model 2	1.00 (Ref)	0.93 (0.77–1.13)	0.85 (0.70–1.02)	0.74 (0.59–0.92)	0.57 (0.44–0.74)	<0.001
**hPDI**						
** MI**						
Median (range)	45 (32, 47)	49 (48, 50)	52 (51, 53)	55 (54, 56)	59 (57, 71)	
Cases (rate, %)	49 (1.91)	48 (1.79)	60 (1.81)	58 (1.95)	65 (2.09)	
Person year	21,222	26,176	34,347	32,070	33,369	
Model 1	1.00 (Ref)	0.75 (0.50–1.11)	0.72 (0.50–1.06)	0.78 (0.53–1.14)	0.83 (0.57–1.20)	0.52
Model 2	1.00 (Ref)	0.71 (0.48–1.06)	0.66 (0.45–0.97)	0.68 (0.46–1.01)	0.63 (0.42–0.95)	0.05
** T2D**						
Median (range)	45 (32, 47)	49 (48, 50)	52 (51, 53)	55 (54, 56)	59 (57, 71)	
Cases (rate, %)	169 (6.52)	198 (7.37)	217 (6.60)	217 (7.24)	250 (8.09)	
Person year	21,053	25,452	33,394	31,488	32,462	
Model 1	1.00 (Ref)	0.90 (0.73–1.10)	0.75 (0.61–0.92)	0.80 (0.65–0.97)	0.88 (0.72–1.07)	0.18
Model 2	1.00 (Ref)	0.89 (0.72–1.09)	0.73 (0.59–0.89)	0.76 (0.62–0.94)	0.81 (0.65–0.99)	0.039
** Stroke**						
Median (range)	45 (32, 47)	49 (48, 50)	52 (51, 53)	55 (54, 56)	59 (57, 73)	
Cases (rate, %)	46 (1.79)	71 (2.63)	91 (2.77)	87 (2.92)	109 (3.51)	
Person year	21,208	26,213	34,095	32,157	33,309	
Model 1	1.00 (Ref)	1.19 (0.82–1.72)	1.18 (0.83–1.69)	1.25 (0.88–1.80)	1.49 (1.06–2.11)	0.019
Model 2	1.00 (Ref)	1.15 (0.79–1.68)	1.22 (0.85–1.75)	1.32 (0.91–1.92)	1.44 (1.00–2.09)	0.038
** All-cause mortality**						
Median (range)	46 (35, 48)	50 (49, 51)	53 (52, 54)	55 (55, 56)	59 (57, 72)	
Cases (rate, %)	212 (8.19)	254 (9.14)	323 (8.55)	218 (9.45)	336 (8.69)	
Person year	21,651	27,288	39,715	24,694	41,138	
Model 1	1.00 (Ref)	0.88 (0.73–1.05)	0.89 (0.75–1.06)	0.96 (0.80–1.16)	0.85 (0.71–1.01)	0.15
Model 2	1.00 (Ref)	0.96 (0.80–1.15)	0.97 (0.81–1.15)	1.10 (0.90–1.33)	1.01 (0.84–1.21)	0.61
**uPDI**						
** MI**						
Median (range)	43 (25, 45)	48 (46, 49)	51 (50, 52)	54 (53, 56)	59 (57, 73)	
Cases (rate, %)	44 (1.59)	43 (1.50)	61 (2.31)	64 (1.99)	68 (2.15)	
Person year	29,429	29,651	26,643	32,487	28,973	
Model 1	1.00 (Ref)	1.25 (0.79–1.99)	2.29 (1.34–3.92)	2.30 (1.18–4.46)	3.14 (1.43–6.90)	0.003
Model 2	1.00 (Ref)	1.62 (1.01–2.60)	3.34 (1.92–5.81)	3.92 (1.97–7.80)	5.90 (2.59–13.48)	<0.001
** T2D**						
Median (range)	43 (25, 45)	48 (46, 49)	51 (50, 52)	54 (53, 56)	59 (57, 72)	
Cases (rate, %)	182 (6.66)	201 (6.99)	188 (7.06)	228 (7.15)	252 (7.90)	
Person year	28,333	29,121	26,328	31,431	28,636	
Model 1	1.00 (Ref)	1.11 (0.91–1.36)	1.12 (0.92–1.38)	1.12 (0.92–1.36)	1.35 (1.11–1.63)	0.003
Model 2	1.00 (Ref)	1.32 (1.07–1.62)	1.50 (1.21–1.85)	1.70 (1.38–2.10)	2.18 (1.75–2.73)	<0.001
** Stroke**						
Median (range)	43 (25, 45)	48 (46, 49)	51 (50, 52)	54 (53, 56)	59 (57, 73)	
Cases (rate, %)	48 (1.73)	75 (2.60)	76 (2.88)	99 (3.11)	106 (3.34)	
Person year	29,384	29,900	26,631	32,005	29,063	
Model 1	1.00 (Ref)	1.81 (1.22–2.68)	2.32 (1.45–3.70)	2.79 (1.60–4.89)	3.44 (1.78–6.65)	<0.001
Model 2	1.00 (Ref)	2.21 (1.48–3.31)	3.15 (1.92–5.16)	4.53 (2.47–8.29)	5.96 (2.86–12.42)	<0.001
** All-cause mortality**						
Median (range)	44 (26, 46)	49 (47, 50)	52 (51, 53)	55 (54, 57)	61 (58, 75)	
Cases (rate, %)	136 (4.72)	168 (5.54)	201 (7.40)	287 (9.00)	551 (15.75)	
Person year	30,806	32,263	28,582	32,757	30,078	
Model 1	1.00 (Ref)	1.53 (1.21–1.95)	2.29 (1.76–2.98)	3.06 (2.28–4.12)	5.74 (4.13–7.99)	<0.001
Model 2	1.00 (Ref)	1.51 (1.18–1.93)	2.37 (1.79–3.15)	3.44 (2.49–4.77)	6.87 (4.70–10.03)	<0.001

*^a^* Data were presented as HR (95% CI) estimated by Cox proportional hazard regression models. Rate was calculated as incidence density. Model 1 adjusted for sex and age. Model 2 further adjusted for BMI, region, urbanization index, educational level, physical activity, baseline hypertension, smoking status, alcohol intake, and total energy intake. Model 2 for stroke also adjusted for the sodium: potassium ratio. Abbreviations: hPDI, healthy PDI; MI, myocardial infarction; PDI, plant-based diet index; Q, quintiles; Ref, reference; T2D, type 2 diabetes; uPDI, unhealthy PDI.

**Table 4:** Associations between plant-based diet indices and GHG emissions in Chinese adults who participated in the China Health and Nutrition Survey 1997–2015 wave *^a^*.

Variables	Quintiles					*p*-Trend
	Q1	Q2	Q3	Q4	Q5	
**PDI**						
** GHG emissions in MI outcome**						
Model 1	7980.34 (7823.34–8140.48) ^a^	5786.53 (5676.29–5898.90) ^b^	4654.57 (4570.18–4740.52) ^c^	4070.74 (3995.01–4147.89) ^d^	3063.62 (3010.60–3117.56) ^e^	<0.001
Model 2	7145.21 (6991.87–7301.90) ^a^	5601.27 (5490.96–5713.80) ^b^	4699.55 (4615.28–4785.37) ^c^	4102.50 (4025.23–4181.24) ^d^	3169.91 (3102.78–3238.49) ^e^	<0.001
** GHG emissions in T2D outcome**						
Model 1	7992.61 (7835.11–8153.28) ^a^	5776.38 (5665.57–5889.36) ^b^	4641.00 (4556.80–4726.76) ^c^	4053.05 (3977.02–4130.53) ^d^	3059.68 (3006.32–3113.99) ^e^	<0.001
Model 2	7161.55 (7007.87–7318.60) ^a^	5592.20 (5481.71–5704.92) ^b^	4683.85 (4599.93–4769.30) ^c^	4081.68 (4004.32–4160.53) ^d^	3152.77 (3085.42–3221.60) ^e^	<0.001
** GHG emissions in Stroke outcome**						
Model 1	7984.09 (7826.72–8144.62) ^a^	5779.23 (5669.24–5891.34) ^b^	4665.25 (4580.76–4751.30) ^c^	4065.69 (3990.00–4142.81) ^d^	3058.42 (3005.46–3112.31) ^e^	<0.001
Model 2	7138.02 (6984.94–7294.46) ^a^	5588.53 (5478.80–5700.46) ^b^	4707.44 (4623.36–4793.05) ^c^	4097.52 (4020.38–4176.14) ^d^	3164.76 (3097.68–3233.28) ^e^	<0.001
** GHG emissions in All-cause mortality outcome**						
Model 1	7572.38 (7438.85–7708.32) ^a^	5575.73 (5473.39–5679.97) ^b^	4398.30 (4326.22–4471.58) ^c^	3878.67 (3813.46–3945.00) ^d^	2908.85 (2858.70–2959.88) ^e^	<0.001
Model 2	6869.88 (6715.92–7027.38) ^a^	5554.91 (5437.02–5675.35) ^b^	4600.12 (4512.89–4689.04) ^c^	3984.27 (3908.03–4062.00) ^d^	2967.33 (2898.21–3038.10) ^e^	<0.001
**hPDI**						
** GHG emissions in MI outcome**						
Model 1	5492.48 (5368.88–5618.93) ^a^	4964.66 (4854.97–5076.83) ^b^	4604.88 (4510.72–4700.99) ^c^	4305.64 (4213.29–4400.02) ^d^	3859.02 (3778.72–3941.03) ^e^	<0.001
Model 2	5890.06 (5770.33–6012.28) ^a^	5287.48 (5184.00–5393.02) ^b^	4735.65 (4649.49–4823.41) ^c^	4319.72 (4238.96–4402.02) ^d^	3772.93 (3703.69–3843.65) ^e^	<0.001
** GHG emissions in T2D outcome**						
Model 1	5494.43 (5370.75–5620.96) ^a^	4969.94 (4859.69–5082.70) ^b^	4593.16 (4498.28–4690.04) ^c^	4326.26 (4233.63–4420.90) ^d^	3837.08 (3756.55–3919.35) ^e^	<0.001
Model 2	5883.47 (5763.72–6005.71) ^a^	5284.92 (5181.13–5390.79) ^b^	4731.05 (4644.42–4819.29) ^c^	4328.31 (4247.44–4410.71) ^d^	3757.92 (3688.50–3828.65) ^e^	<0.001
** GHG emissions in Stroke outcome**						
Model 1	5491.01 (5367.37–5617.48) ^a^	4939.59 (4830.59–5051.05) ^b^	4618.78 (4524.04–4715.50) ^c^	4310.81 (4218.45–4405.20) ^d^	3857.34 (3777.07–3939.31) ^e^	<0.001
Model 2	5883.62 (5764.25–6005.47) ^a^	5272.63 (5169.79–5377.52) ^b^	4738.02 (4651.66–4825.98) ^c^	4324.43 (4243.92–4406.46) ^d^	3771.64 (3702.55–3842.01) ^e^	<0.001
** GHG emissions in All-cause mortality outcome**						
Model 1	5649.64 (5527.32–5774.67) ^a^	5119.22 (5012.24–5228.48) ^b^	4640.30 (4557.01–4725.11) ^c^	4275.37 (4177.44–4375.60) ^d^	3720.16 (3654.15–3787.37) ^e^	<0.001
Model 2	5652.94 (5531.43–5777.12) ^a^	5109.66 (5004.40–5217.14) ^b^	4620.38 (4533.98–4708.42) ^c^	4201.77 (4111.71–4293.81) ^d^	3729.58 (3661.81–3798.61) ^e^	<0.001
**uPDI**						
** GHG emissions in MI outcome**						
Model 1	7010.53 (6882.53–7140.91) ^a^	5924.69 (5816.81–6034.56) ^b^	4939.79 (4846.91–5034.46) ^c^	3992.65 (3923.78–4062.73) ^d^	2771.25 (2723.58–2819.74) ^e^	<0.001
Model 2	5934.54 (5821.30–6049.97) ^a^	5428.36 (5328.03–5530.59) ^b^	4837.28 (4746.71–4929.57) ^c^	4211.91 (4136.71–4288.48) ^d^	3322.78 (3260.61–3386.13) ^e^	<0.001
** GHG emissions in T2D outcome**						
Model 1	7040.83 (6910.55–7173.58) ^a^	5941.05 (5832.66–6051.45) ^b^	4985.54 (4891.87–5081.00) ^c^	3991.14 (3921.68–4061.83) ^d^	2774.46 (2726.73–2823.01) ^e^	<0.001
Model 2	5943.76 (5829.02–6060.76) ^a^	5429.03 (5328.53–5531.42) ^b^	4878.28 (4786.88–4971.43) ^c^	4200.06 (4124.76–4276.74) ^d^	3332.55 (3270.15–3396.15) ^e^	<0.001
** GHG emissions in Stroke outcome**						
Model 1	7004.90 (6876.80–7135.38) ^a^	5905.85 (5798.67–6015.01) ^b^	4947.37 (4854.28–5042.24) ^c^	3998.63 (3929.23–4069.27) ^d^	2770.29 (2722.74–2818.68) ^e^	<0.001
Model 2	5924.85 (5811.78–6040.12) ^a^	5410.90 (5311.23–5512.43) ^b^	4837.44 (4746.96–4929.64) ^c^	4216.54 (4141.04–4293.41) ^d^	3329.24 (3267.07–3392.60) ^e^	<0.001
** GHG emissions in All-cause mortality outcome**						
Model 1	7061.19 (6938.46–7186.09) ^a^	5914.73 (5814.52–6016.67) ^b^	4878.86 (4791.53–4967.78) ^c^	3965.54 (3900.01–4032.17) ^d^	2741.25 (2697.98–2785.21) ^e^	<0.001
Model 2	5733.02 (5616.85–5851.57) ^a^	5214.87 (5113.37–5318.39) ^b^	4617.22 (4524.76–4711.58) ^c^	4046.88 (3969.22–4126.06) ^d^	3156.06 (3091.97–3221.48) ^e^	<0.001

*^a^* Data were presented as Lsmeans (95% CL) estimated by multiple linear regression models, values with superscript letters a, b, c, d, and e were significantly different from each other (*p* < 0.05). Model 1 adjusted for sex and age. Model 2 further adjusted for BMI, region, urbanization index, educational level, physical activity, baseline hypertension, smoking status, alcohol intake, and total energy intake. N = 14,652 for cohort A with cardiometabolic diseases as primary outcomes, and N = 15,318 for cohort B with all-cause mortality as the primary outcome. Abbreviations: GHG, greenhouse gas; hPDI, healthy PDI; MI, myocardial infarction; PDI, plant-based diet index; Q, quintiles; T2D, type 2 diabetes; uPDI, unhealthy PDI.

**Table 5:** Associations between plant-based diet indices and risk of MI, T2D, stroke, and all-cause mortality in sensitivity analysis excluding participants who were diagnosed with MI, T2D, stroke, or died during the first two years of follow-up *^a^*.

Variables	Quintiles					*p*-Trend
	Q1	Q2	Q3	Q4	Q5	
**PDI**						
** MI (N = 14,644)**						
Median (range)	37 (23, 40)	43 (41, 45)	48 (46, 50)	53 (51, 55)	59 (56, 72)	
Cases (rate, %)	47 (1.71)	44 (1.54)	68 (2.22)	57 (2.09)	56 (1.72)	
Person year	28,555	31,079	33,009	26,883	27,641	
Model 1	1.00 (Ref)	0.76 (0.50–1.14)	1.01 (0.70–1.47)	0.90 (0.61–1.34)	0.83 (0.55–1.25)	0.64
Model 2	1.00 (Ref)	0.69 (0.45–1.05)	0.84 (0.56–1.25)	0.68 (0.42–1.09)	0.47 (0.27–0.84)	0.032
** T2D (N = 14,639)**						
Median (range)	37 (23, 40)	43 (41, 45)	48 (46, 50)	53 (51, 55)	59 (56, 72)	
Cases (rate, %)	223 (8.08)	204 (7.17)	219 (7.08)	190 (7.01)	202 (6.25)	
Person year	28,063	30,313	32,497	26,207	26,745	
Model 1	1.00 (Ref)	0.79 (0.65–0.95)	0.74 (0.62–0.90)	0.75 (0.61–0.91)	0.79 (0.64–0.96)	0.018
Model 2	1.00 (Ref)	0.71 (0.58–0.86)	0.56 (0.46–0.69)	0.46 (0.36–0.59)	0.34 (0.25–0.45)	<0.001
** Stroke (N = 14,642)**						
Median (range)	37 (23, 40)	43 (41, 45)	48 (46, 50)	53 (51, 55)	59 (56, 72)	
Cases (rate, %)	59 (2.16)	73 (2.55)	98 (3.18)	78 (2.86)	86 (2.65)	
Person year	28,390	31,153	33,116	26,696	27,608	
Model 1	1.00 (Ref)	1.01 (0.72–1.42)	1.24 (0.90–1.72)	1.17 (0.83–1.65)	1.24 (0.88–1.76)	0.14
Model 2	1.00 (Ref)	0.90 (0.63–1.28)	0.93 (0.66–1.33)	0.75 (0.50–1.13)	0.62 (0.39–1.01)	0.06
** All-cause mortality (N = 15,293)**						
Median (range)	38 (25, 41)	44 (42, 45)	48 (46, 50)	53 (51, 56)	60 (57, 75)	
Cases (rate, %)	210 (7.18)	211 (7.82)	282 (8.32)	313 (9.72)	302 (9.87)	
Person year	30,520	29,729	37,533	31,827	24,849	
Model 1	1.00 (Ref)	0.96 (0.79–1.16)	0.88 (0.74–1.06)	0.96 (0.80–1.14)	1.01 (0.84–1.21)	0.80
Model 2	1.00 (Ref)	0.92 (0.76–1.12)	0.84 (0.69–1.01)	0.73 (0.59–0.92)	0.57 (0.44–0.74)	<0.001
**hPDI**						
** MI (N = 14,644)**						
Median (range)	45 (32, 47)	49 (48, 50)	52 (51, 53)	55 (54, 56)	59 (57, 71)	
Cases (rate, %)	48 (1.87)	45 (1.68)	59 (1.78)	57 (1.92)	63 (2.03)	
Person year	21,240	26,126	34,405	32,036	33,360	
Model 1	1.00 (Ref)	0.71 (0.48–1.07)	0.72 (0.49–1.05)	0.77 (0.53–1.14)	0.81 (0.56–1.18)	0.52
Model 2	1.00 (Ref)	0.68 (0.45–1.02)	0.65 (0.44–0.96)	0.67 (0.45–1.00)	0.62 (0.41–0.93)	0.05
** T2D (N = 14,639)**						
Median (range)	45 (32, 47)	49 (48, 50)	52 (51, 53)	55 (54, 56)	59 (57, 71)	
Cases (rate, %)	167 (6.47)	194 (7.20)	215 (6.55)	215 (7.17)	240 (8.01)	
Person year	20,993	25,544	33,364	31,520	32,403	
Model 1	1.00 (Ref)	0.88 (0.72–1.08)	0.75 (0.61–0.92)	0.79 (0.65–0.97)	0.87 (0.72–1.06)	0.19
Model 2	1.00 (Ref)	0.87 (0.71–1.07)	0.72 (0.59–0.89)	0.75 (0.61–0.93)	0.80 (0.65–0.99)	0.040
** Stroke (N = 14,642)**						
Median (range)	45 (32, 47)	49 (48, 50)	52 (51, 53)	55 (54, 56)	59 (57, 71)	
Cases (rate, %)	44 (1.71)	69 (2.56)	87 (2.64)	88 (2.95)	106 (3.41)	
Person year	21,178	26,195	34,139	32,131	33,320	
Model 1	1.00 (Ref)	1.20 (0.82–1.75)	1.17 (0.81–1.68)	1.31 (0.91–1.88)	1.50 (1.05–2.13)	0.017
Model 2	1.00 (Ref)	1.16 (0.80–1.70)	1.20 (0.83–1.74)	1.39 (0.96–2.03)	1.46 (1.00–2.12)	0.030
** All-cause mortality (N = 15,293)**						
Median (range)	46 (35, 48)	50 (49, 51)	53 (52, 54)	55 (55, 56)	59 (57, 72)	
Cases (rate, %)	207 (8.02)	249 (8.98)	320 (8.48)	212 (9.21)	330 (8.55)	
Person year	21,647	27,281	39,713	24,688	41,131	
Model 1	1.00 (Ref)	0.88 (0.73–1.06)	0.90 (0.75–1.07)	0.95 (0.79–1.15)	0.85 (0.71–1.01)	0.15
Model 2	1.00 (Ref)	0.96 (0.80–1.16)	0.98 (0.82–1.18)	1.10 (0.90–1.34)	1.03 (0.85–1.24)	0.52
**uPDI**						
** MI (N = 14,644)**						
Median (range)	43 (25, 45)	48 (46, 49)	51 (50, 52)	54 (53, 56)	59 (57, 73)	
Cases (rate, %)	43 (1.55)	41 (1.43)	58 (2.21)	64 (1.99)	66 (2.09)	
Person year	29,443	29,666	26,615	32,460	28,984	
Model 1	1.00 (Ref)	1.30 (0.81–2.09)	2.49 (1.42–4.36)	2.79 (1.39–5.58)	3.89 (1.70–8.91)	<0.001
Model 2	1.00 (Ref)	1.65 (1.02–2.69)	3.50 (1.97–6.24)	4.60 (2.24–9.46)	6.94 (2.92–16.53)	<0.001
** T2D (N = 14,639)**						
Median (range)	43 (25, 45)	48 (46, 49)	51 (50, 52)	54 (53, 56)	59 (57, 72)	
Cases (rate, %)	179 (6.56)	198 (6.89)	185 (6.95)	227 (7.12)	249 (7.81)	
Person year	28,321	29,122	26,315	31,435	28,632	
Model 1	1.00 (Ref)	1.11 (0.91–1.36)	1.13 (0.92–1.38)	1.14 (0.93–1.38)	1.36 (1.12–1.65)	0.003
Model 2	1.00 (Ref)	1.32 (1.08–1.62)	1.51 (1.22–1.87)	1.73 (1.39–2.13)	2.20 (1.76–2.76)	<0.001
** Stroke (N = 14,642)**						
Median (range)	43 (25, 45)	48 (46, 49)	51 (50, 52)	54 (53, 56)	59 (57, 73)	
Cases (rate, %)	46 (1.66)	73 (2.54)	75 (2.84)	97 (3.04)	103 (3.25)	
Person year	29,405	29,861	26,638	32,005	29,054	
Model 1	1.00 (Ref)	1.88 (1.26–2.81)	2.49 (1.53–4.03)	3.03 (1.69–5.43)	3.78 (1.89–7.54)	<0.001
Model 2	1.00 (Ref)	2.32 (1.54–3.51)	3.45 (2.07–5.74)	5.07 (2.70–9.52)	6.80 (3.16–14.63)	<0.001
** All-cause mortality (N = 15,293)**						
Median (range)	44 (26, 46)	49 (47, 50)	52 (51, 53)	55 (54, 57)	61 (58, 75)	
Cases (rate, %)	135 (4.69)	163 (5.38)	197 (7.27)	281 (8.83)	542 (15.53)	
Person year	30,805	32,258	28,578	32,750	30,068	
Model 1	1.00 (Ref)	1.52 (1.19–1.93)	2.31 (1.77–3.02)	3.13 (2.31–4.24)	5.99 (4.27–8.40)	<0.001
Model 2	1.00 (Ref)	1.47 (1.15–1.89)	2.32 (1.73–3.10)	3.33 (2.36–4.71)	6.65 (4.42–10.01)	<0.001

*^a^* Data were presented as HR (95% CI) estimated by Cox proportional hazard regression models. Rate was calculated as incidence density. Model 1 adjusted for sex and age. Model 2 further adjusted for BMI, region, urbanization index, educational level, physical activity, baseline hypertension, smoking status, alcohol intake, and total energy intake. Model 2 for stroke also adjusted for the sodium: potassium ratio. Abbreviations: hPDI, healthy PDI; MI, myocardial infarction; PDI, plant-based diet index; Q, quintiles; Ref, reference; T2D, type 2 diabetes; uPDI, unhealthy PDI.

**Table 6:** Associations between plant-based diet indices and risk of all-cause mortality (N = 14,676) in sensitivity analysis excluding participants who were diagnosed with CMDs or tumors or took medicines to treat CMDs at baseline *^a^*.

Variables	Quintiles					*p*-Trend
	Q1	Q2	Q3	Q4	Q5	
**PDI**						
Median (range)	38 (25, 40)	43 (41, 45)	48 (46, 49)	52 (50, 54)	58 (55, 73)	
Cases (rate, %)	172 (6.86)	257 (7.81)	251 (9.11)	255 (9.00)	312 (9.49)	
Person year	26,490	36,741	30,157	28,155	28,706	
Model 1	1.00 (Ref)	0.99 (0.82–1.20)	1.01 (0.83–1.22)	0.93 (0.77–1.14)	0.98 (0.81–1.20)	0.75
Model 2	1.00 (Ref)	1.01 (0.82–1.23)	0.95 (0.77–1.18)	0.79 (0.63–1.00)	0.67 (0.52–0.88)	<0.001
**hPDI**						
Median (range)	44 (32, 46)	48 (47, 49)	51 (50, 52)	54 (53, 55)	58 (56, 71)	
Cases (rate, %)	209 (8.62)	248 (9.59)	278 (8.17)	262 (8.27)	250 (8.08)	
Person year	19,901	25,707	35,961	35,042	33,638	
Model 1	1.00 (Ref)	0.90 (0.75–1.08)	0.89 (0.75–1.07)	0.88 (0.74–1.06)	0.84 (0.70–1.01)	0.08
Model 2	1.00 (Ref)	0.93 (0.77–1.13)	1.01 (0.84–1.22)	1.04 (0.86–1.27)	1.03 (0.84–1.25)	0.50
**uPDI**						
Median (range)	45 (28, 47)	50 (48, 51)	53 (52, 55)	57 (56, 59)	63 (60, 78)	
Cases (rate, %)	107 (4.21)	133 (4.94)	232 (7.11)	257 (9.15)	518 (15.37)	
Person year	27,722	29,516	34,994	29,276	28,741	
Model 1	1.00 (Ref)	1.46 (1.11–1.90)	2.44 (1.84–3.24)	3.31 (2.38–4.61)	6.08 (4.21–8.78)	<0.001
Model 2	1.00 (Ref)	1.55 (1.17–2.04)	2.77 (2.03–3.77)	4.21 (2.89–6.12)	8.93 (5.74–13.91)	<0.001

*^a^* Data were presented as HR (95% CI) estimated by Cox proportional hazard regression models. Rate was calculated as incidence density. Model 1 adjusted for sex and age. Model 2 further adjusted for BMI, region, urbanization index, educational level, physical activity, baseline hypertension, smoking status, alcohol intake, and total energy intake. Abbreviations: CMDs, cardiometabolic diseases, including myocardial infarction, type 2 diabetes mellitus, and stroke; hPDI, healthy PDI; PDI, plant-based diet index; Q, quintiles; Ref, reference; uPDI, unhealthy PDI.

## Data Availability

The data that support the findings of this study are available from the corresponding author upon reasonable request. The data are not publicly available due to it is part of an ongoing study.

## Supplementary Materials

The following supporting information can be downloaded at: https://www.mdpi.com/article/10.3390/nu17071152/s1, Figure S1: Participant flow diagram in China Health and Nutrition Survey 1997–2015 wave for new-onset CMDs (A) and all-cause mortality (B) as primary outcomes; Table S1: STROBE Statement; Table S2: Scoring criteria for each plant-based dietary index and food items of each food group (from 1997–2011 CHNS food frequency questionnaire); Table S3: Baseline sociodemographic, anthropometric and lifestyle characteristics of 15,318 Chinese adults in cohort B who participated in the China Health and Nutrition Survey 1997–2015 wave based on quintiles of plant-based diet indices; Table S4: Baseline daily intakes of nutrients of 14,652 Chinese adults in cohort A who participated in the China Health and Nutrition Survey 1997–2015 wave based on quintiles of plant-based diet indices; Table S5: Baseline daily intakes of nutrients and food groups of 15,318 Chinese adults in cohort B who participated in the China Health and Nutrition Survey 1997–2015 wave based on quintiles of plant-based diet indices; Figure S2: Associations between plant-based diet indices and risk of MI (N = 14,652) in Chinese adults who participated in the China Health and Nutrition Survey 1997–2015 wave, stratified by age, sex, BMI, region and baseline hypertension history (A: PDI and risk of MI, B: hPDI and risk of MI, C: uPDI and risk of MI); Table S6: Associations between healthy plant-based diet index and risk of MI, T2D, stroke (N = 14,652) and all-cause mortality (N = 15,318) in Chinese adults who participated in the China Health and Nutrition Survey 1997–2015 wave, stratified by age, sex, BMI, region and baseline hypertension history; Table S7: Associations between healthy plant-based diet index and risk of MI, T2D, stroke (N = 14,652) and all-cause mortality (N = 15,318) after positive coding for dairy products and fish and seafood; Table S8: Associations between revised plant-based diet indices after excluding each food group and risk of MI, T2D, stroke (N = 14,652) and all-cause mortality (N = 15,318) in Chinese adults who participated in the China Health and Nutrition Survey 1997–2015 wave.
