# Supplementary material for: Plant-Based Diet Indices with Greenhouse Gas Emissions and Risk of Cardiometabolic Diseases and All-Cause Mortality: Longitudinal China Cohort Study
Source: Nutrients. 2025 Mar 26;17(7):1152. doi: 10.3390/nu17071152 (PMC11990547; doi:10.3390/nu17071152)
Supplement: Supplementary file 1 [file nutrients-17-01152-s001.zip › nutrients-3528619-supplementary.pdf]

Associations between adherence to plant-based diets with greenhouse gas emissions and risk of cardiometabolic diseases and all-cause mortality

Supplementary Materials

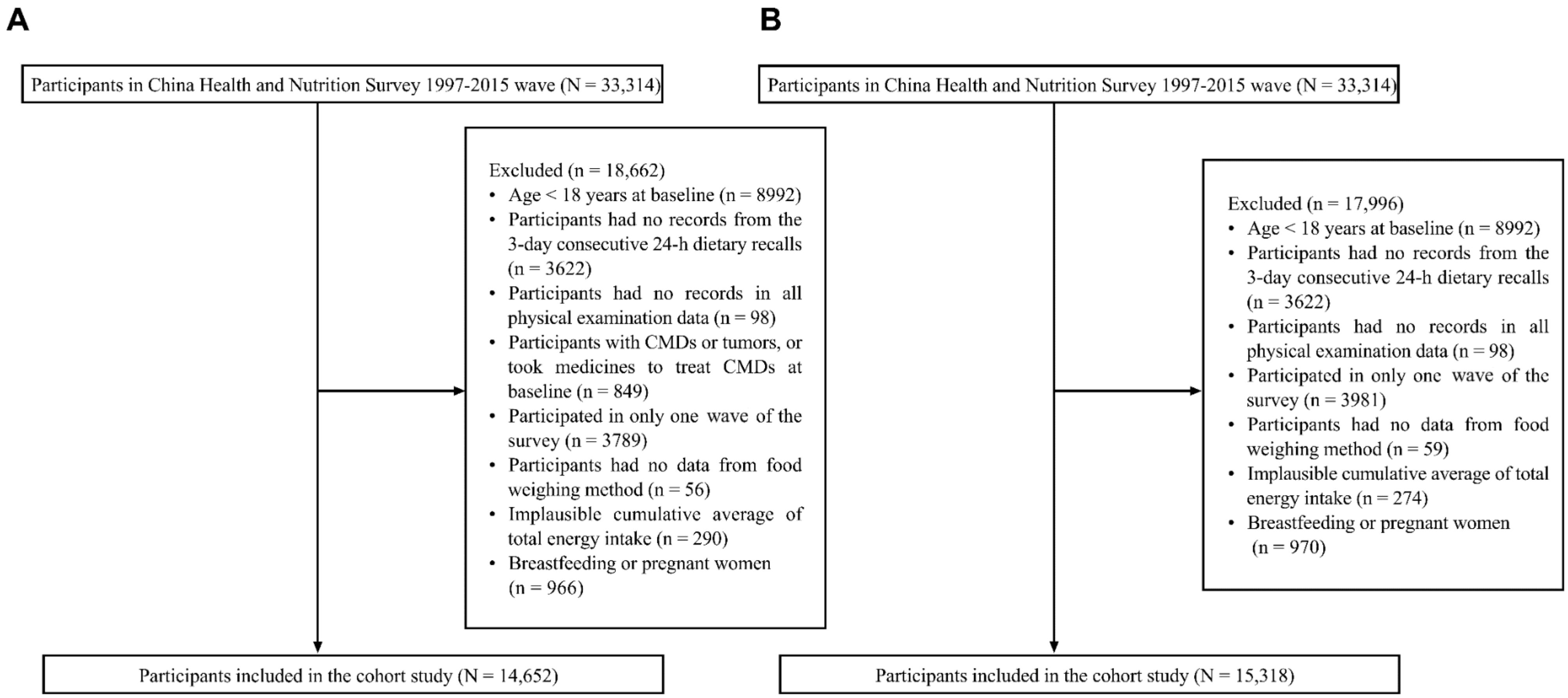

Figure S1. Participant flow diagram in China Health and Nutrition Survey 1997-2015 wave for new-onset CMDs (A) and all-cause mortality (B) as primary outcomes. Abbreviations: CMD, cardiometabolic disease.

Table S1. STROBE Statement.

	Item No	Recommendation	Page No.
**Title and abstract**	1	(a) Indicate the study's design with a commonly used term in the title or the abstract	P1
		(b) Provide in the abstract an informative and balanced summary of what was done and what was found	P1
**Introduction**			
Background/rationale	2	Explain the scientific background and rationale for the investigation being reported	P1-2
Objectives	3	State specific objectives, including any prespecified hypotheses	P2
**Methods**			
Study design	4	Present key elements of study design early in the paper	P3-4
Setting	5	Describe the setting, locations, and relevant dates, including periods of recruitment, exposure, follow-up, and data collection	P3-4
Participants	6	(a) Give the eligibility criteria, and the sources and methods of selection of participants. Describe methods of follow-up	P3-4
		(b) For matched studies, give matching criteria and number of exposed and unexposed	N/A
Variables	7	Clearly define all outcomes, exposures, predictors, potential confounders, and effect modifiers. Give diagnostic criteria, if applicable	P4-6
Data sources/ measurement	8*	For each variable of interest, give sources of data and details of methods of assessment (measurement). Describe comparability of assessment methods if there is more than one group	P3-6
Bias	9	Describe any efforts to address potential sources of bias	N/A
Study size	10	Explain how the study size was arrived at	N/A
Quantitative variables	11	Explain how quantitative variables were handled in the analyses. If applicable, describe which groupings were chosen and why	N/A
Statistical methods	12	(a) Describe all statistical methods, including those used to control for confounding	P6-7
		(b) Describe any methods used to examine subgroups and interactions	P6-7
		(c) Explain how missing data were addressed	N/A
		(d) If applicable, explain how loss to follow-up was addressed	N/A
		(e) Describe any sensitivity analyses	P6-7
**Results**			
Participants	13*	(a) Report numbers of individuals at each stage of study—eg numbers potentially eligible, examined for eligibility, confirmed eligible, included in the study, completing follow-up, and analysed	P3-4
		(b) Give reasons for non-participation at each stage	P3-4
		(c) Consider use of a flow diagram	SuppleP2
Descriptive data	14*	(a) Give characteristics of study participants (eg demographic, clinical, social) and information on exposures and potential confounders	P7-9

		(b) Indicate number of participants with missing data for each variable of interest	N/A
		(c) Summarise follow-up time (eg, average and total amount)	P7
Outcome data	15*	Report numbers of outcome events or summary measures over time	N/A
Main results	16	(a) Give unadjusted estimates and, if applicable, confounder-adjusted estimates and their precision (eg, 95% confidence interval). Make clear which confounders were adjusted for and why they were included	P10-17
		(b) Report category boundaries when continuous variables were categorized	P6-7
		(c) If relevant, consider translating estimates of relative risk into absolute risk for a meaningful time period	N/A
Other analyses	17	Report other analyses done—eg analyses of subgroups and interactions, and sensitivity analyses	P13-17
**Discussion**			
Key results	18	Summarise key results with reference to study objectives	P17
Limitations	19	Discuss limitations of the study, taking into account sources of potential bias or imprecision. Discuss both direction and magnitude of any potential bias	P19
Interpretation	20	Give a cautious overall interpretation of results considering objectives, limitations, multiplicity of analyses, results from similar studies, and other relevant evidence	P17-19
Generalisability	21	Discuss the generalisability (external validity) of the study results	P19
**Other information**			
Funding	22	Give the source of funding and the role of the funders for the present study and, if applicable, for the original study on which the present article is based	P20

* Give information separately for exposed and unexposed groups.

Table S2. Scoring criteria for each plant-based dietary index and food items of each food group (from 1997-2011 CHNS food frequency questionnaire).

		PDI	hPDI	uPDI
**Healthy plant-based food groups**				
Whole grains	Wheat, wheat germ, bran, black rice, brown rice, corn, barley, millet, yellow rice, cereal, oatmeal, other grains	Positive score	Positive score	Reverse score
Fruits	kernel and stone fruits, berry fruit, citrus, tropical and subtropical fruits, melons	Positive score	Positive score	Reverse score
Vegetables	Root vegetables, fresh beans, eggplant, garlic and onion, young stems and leaves, aquatic vegetables, yams or sweet potatoes, wild vegetables, mushrooms and algae	Positive score	Positive score	Reverse score
Nuts	Peanuts and nuts	Positive score	Positive score	Reverse score
Legumes	String beans, tofu or soybeans, beans or lentils, peas or lima beans	Positive score	Positive score	Reverse score
Vegetable oils	Vegetable oil used for cooking	Positive score	Positive score	Reverse score
Tea and coffee	Tea, coffee	Positive score	Positive score	Reverse score
**Unhealthy plant-based food groups**				
Fruit juices	Orange juice, lemon juice, plum juice, other juices	Positive score	Reverse score	Positive score
Refined grains	Noodles, white rice, bread, biscuits, and other leisure foods, flour, instant noodles, pancakes, steamed buns, dumplings, rice cake	Positive score	Reverse score	Positive score
Potatoes and starch	Potatoes, chips, starch	Positive score	Reverse score	Positive score
Sugar-sweetened beverages	Carbonated beverages, solid beverages, milk beverages, plant protein beverages, other beverages.	Positive score	Reverse score	Positive score
Sweets and desserts	Cakes, desserts, mooncakes, sugars, preserves, jams	Positive score	Reverse score	Positive score
**Animal-based food groups**				
Animal fat	lard, butter or mutton tallow	Reverse score	Reverse score	Reverse score
Dairy	Milk, yogurt, ice cream, cheese	Reverse score	Reverse score	Reverse score
Eggs	Eggs	Reverse score	Reverse score	Reverse score
Fish or seafood	Fish, shrimp, crab, shellfish and mollusks	Reverse score	Reverse score	Reverse score
Meat	Pork, beef, mutton or lamb, other red meat, processed meats, chicken and other poultry	Reverse score	Reverse score	Reverse score

Abbreviations: CHNS, China Health and Nutrition Survey; hPDI, healthy PDI; PDI, plant-based diet index; uPDI, unhealthy PDI.

Table S3. Baseline sociodemographic, anthropometric and lifestyle characteristics of 15,318 Chinese adults in cohort B who participated in the China Health and Nutrition Survey 1997-2015 wave based on quintiles of plant-based diet indices [a].

Variables	All	Quintiles of PDI			P^{b}	Quintiles of hPDI			P^{b}	Quintiles of uPDI			P^{b}
		Q1	Q3	Q5		Q1	Q3	Q5		Q1	Q3	Q5	
N	15,318	2930	2919	3502		2705	3390	3251		2884	3093	3171	
PDI	47 (43, 51)	39 (37, 40)	46 (45, 47)	55 (53, 57)	<0.001	49 (44, 53)	46 (41, 51)	47 (44, 50)	<0.001	45 (41, 49)	46 (42, 50)	49 (46, 53)	<0.001
hPDI	52 (47, 57)	51 (48, 55)	54 (49, 58)	49 (46, 54)	<0.001	43 (41, 44)	51 (51, 52)	60 (59, 62)	<0.001	55 (51, 59)	52 (47, 56)	49 (45, 54)	<0.001
uPDI	54 (50, 58)	51 (48, 55)	54 (50, 57)	56 (52, 60)	<0.001	56 (53, 60)	54 (50, 58)	52 (47, 56)	<0.001	46 (44, 47)	53 (52, 54)	61 (60, 63)	<0.001
Age, years	46 ± 15	48 ± 16	46 ± 15	43 ± 14	<0.001	42 ± 13	45 ± 15	50 ± 16	<0.001	49 ± 15	46 ± 15	43 ± 15	<0.001
Female, N (%)	7558 (49.3)	1640 (56.0)	1579 (54.1)	1304 (37.2)	<0.001	828 (30.6)	1660 (49.0)	2143 (65.9)	0.19	1599 (55.4)	1457 (47.1)	1459 (46.0)	<0.001
BMI, kg/m^2	22.6 (20.6, 25.1)	22.5 (20.5, 24.9)	22.6 (20.6, 25.1)	22.5 (20.6, 25.0)	<0.001	22.2 (20.3, 24.5)	22.5 (20.6, 25.0)	23.2 (20.9, 25.7)	<0.001	23.5 (21.3, 25.8)	22.6 (20.6, 25.0)	21.8 (20.1, 24.0)	<0.001
SBP, mmHg	120.0 (110.0, 130.0)	120.0 (110.0, 130.7)	120.0 (110.0, 130.0)	120.0 (110.0, 130.0)	<0.001	118.7 (110.0, 126.7)	120.0 (110.0, 130.0)	120.0 (110.0, 134.7)	<0.001	120.7 (110.0, 131.7)	120.0 (110.0, 130.0)	120.0 (110.0, 128.0)	<0.001
DBP, mmHg	79.3 (70.0, 84.0)	78.7 (70.0, 83.8)	78.7 (70.0, 83.3)	80.0 (70.0, 84.7)	<0.001	78.7 (70.0, 83.3)	79.0 (70.0, 84.0)	79.3 (70.0, 85.0)	<0.001	79.3 (70.7, 84.7)	79.3 (70.0, 84.0)	78.7 (70.0, 82.0)	<0.001
Education level, N (%)					<0.001				<0.001				<0.001
Primary	7186 (46.9)	1333 (45.5)	1373 (47.0)	1724 (49.2)		1242 (45.9)	1562 (46.1)	1574 (48.4)		833 (28.9)	1442 (46.6)	1946 (61.4)	
Middle	4353 (28.4)	739 (25.2)	813 (27.9)	1096 (31.3)		878 (32.5)	976 (28.8)	840 (25.9)		800 (27.7)	892 (28.9)	878 (27.7)	
High	3779 (24.7)	858 (29.3)	733 (25.1)	682 (19.5)		585 (21.6)	852 (25.1)	837 (25.7)		1251 (43.4)	759 (24.5)	347 (10.9)	
Urbanization index, N (%)					<0.001				<0.001				<0.001
Low	5085 (33.2)	518 (17.7)	896 (30.7)	1746 (49.9)		1107 (40.9)	1126 (33.2)	853 (26.2)		180 (6.2)	867 (28.0)	2081 (65.6)	

Medium	5089 (33.2)	1167 (39.8)	996 (34.1)	949 (27.1)		996 (36.8)	1112 (32.8)	1015 (31.3)		755 (26.2)	1255 (40.6)	785 (24.8)	
High	5144 (33.6)	1245 (42.5)	1027 (35.2)	807 (23.0)		602 (22.3)	1152 (34.0)	1383 (42.5)		1949 (67.6)	971 (31.4)	305 (9.6)	
Region, N (%)					<0.001				<0.001				<0.001
Southern	8918 (58.2)	2167 (74.0)	1710 (58.6)	1554 (44.4)		1772 (65.5)	2014 (59.4)	1590 (48.9)		1839 (63.8)	1928 (62.3)	1464 (46.2)	
Northern	6400 (41.8)	763 (26.0)	1209 (41.4)	1948 (55.6)		933 (34.5)	1376 (40.6)	1661 (51.1)		1045 (36.2)	1165 (37.7)	1707 (53.8)	
Currently smoking, N (%)	4813 (31.4)	787 (26.9)	845 (28.9)	1384 (39.5)	<0.001	1195 (44.2)	1086 (32.0)	680 (20.9)	0.57	710 (24.6)	984 (31.8)	1153 (36.4)	<0.001
Currently drinking alcohol, N (%)	5600 (36.6)	945 (32.3)	983 (33.7)	1537 (43.9)	<0.001	1324 (48.9)	1263 (37.3)	839 (25.8)	0.35	1066 (37.0)	1116 (36.1)	1131 (35.7)	<0.001
Physical activity status, N (%)					<0.001				<0.001				<0.001
Low	5053 (33.0)	1156 (39.5)	1039 (35.6)	876 (25.0)		645 (23.8)	1055 (31.1)	1463 (45.0)		1195 (41.4)	1038 (33.6)	721 (22.7)	
Medium	5158 (33.7)	1138 (38.8)	955 (32.7)	1005 (28.7)		824 (30.5)	1179 (34.8)	1089 (33.5)		1204 (41.8)	1132 (36.6)	764 (24.1)	
High	5107 (33.3)	636 (21.7)	925 (31.7)	1621 (46.3)		1236 (45.7)	1156 (34.1)	699 (21.5)		485 (16.8)	923 (29.8)	1686 (53.2)	

[a] Continuous variables were presented as mean ± SD or median (P25, P75), and the categorical variables were presented as N (%). [b] Kruskal-Wallis rank-sum analysis was used in continuous variables and Chi-Square test was used in the categorical variables to test significant differences across different quintiles of plant-based diet indices. Abbreviations: BMI, body mass index; DBP, diastolic blood pressure; hPDI, healthy PDI; PDI, plant-based diet index; Q, quintiles; SBP, systolic blood pressure; SD, standard deviation; uPDI, unhealthy PDI.

Table S4. Baseline daily intakes of nutrients of 14,652 Chinese adults in cohort A who participated in the China Health and Nutrition Survey 1997-2015 wave based on quintiles of plant-based diet indices [a].

Variables	All	Quintiles of PDI			P [b]	Quintiles of hPDI			P [b]	Quintiles of uPDI			P [b]
		Q1	Q3	Q5		Q1	Q3	Q5		Q1	Q3	Q5	
Total energy, kcal	2219.8 ± 735.8	1940.6 ± 458.4	2062.2 ± 688.9	2853.5 ± 713.7	<0.001	3037.7 ± 675.0	2154.8 ± 577.1	1711.4 ± 433.0	<0.001	1841.5 ± 626.3	2245.5 ± 743.9	2531.6 ± 636.2	<0.001
Carbohydrate, % E	56.2 ± 13.4	51.1 ± 11.4	56.9 ± 13.9	60.1 ± 12.9	<0.001	57.6 ± 13.3	56.1 ± 13.3	55.2 ± 13.3	<0.001	44.6 ± 9.9	55.5 ± 10.7	68.3 ± 9.5	<0.001
Protein, % E	12.3 ± 3.0	13.6 ± 3.3	12.2 ± 3.0	11.4 ± 2.4	<0.001	11.8 ± 2.9	12.6 ± 3.1	12.1 ± 2.8	<0.001	14.5 ± 3.2	11.9 ± 2.6	10.9 ± 2.2	<0.001
Fat, % E	29.7 ± 12.8	33.8 ± 11.0	29.2 ± 13.3	26.4 ± 12.5	<0.001	28.5 ± 12.7	29.7 ± 12.5	31.0 ± 13.0	<0.001	39.0 ± 10.0	30.8 ± 11.0	19.2 ± 9.7	<0.001
SFA, % E	7.0 ± 3.6	9.2 ± 3.5	6.8 ± 3.5	5.3 ± 2.9	<0.001	7.7 ± 4.1	7.0 ± 3.5	6.5 ± 3.3	<0.001	9.2 ± 2.8	7.4 ± 3.5	4.3 ± 2.7	<0.001
MUFA, % E	11.8 ± 6.2	14.2 ± 5.4	11.7± 6.4	9.6 ± 5.8	<0.001	12.0 ± 6.3	11.8 ± 6.1	11.7 ± 6.4	0.12	15.4 ± 5.5	12.5 ± 5.6	7.1 ± 4.5	<0.001
PUFA, % E	7.5 ± 4.8	6.6 ± 4.1	7.3 ± 4.8	8.2 ± 5.0	<0.001	5.5 ± 4.0	7.4 ± 4.6	9.2 ± 5.0	<0.001	10.1 ± 4.6	7.4 ± 4.7	5.2 ± 3.9	<0.001
Cholesterol, mg	156.9 ± 180.1	250.9 ± 210.5	141.3 ± 162.8	108.9 ± 158.4	<0.001	236.2 ± 238.2	164.7 ± 177.2	90.5 ± 116.5	<0.001	209.9 ± 183.5	175.0 ± 170.0	70.8 ± 116.0	<0.001
Dietary fiber, g	11.7 ± 8.8	8.3 ± 6.5	10.5 ± 6.7	17.5 ± 10.6	<0.001	13.9 ± 11.6	11.4 ± 8.8	10.5 ± 5.9	<0.001	11.0 ± 8.0	11.0 ± 8.7	13.8 ± 10.1	<0.001
Vitamin A, RE	470.0 ± 791.1	583.8 ± 1359.9	458.5 ± 720.9	414.1 ± 623.1	<0.001	610.2 ± 1375.8	448.4 ± 482.8	383.0 ± 529.8	<0.001	616.5 ± 710.5	474.6 ± 625.4	310.0 ± 412.7	<0.001
Vitamin B_1, mg	1.0 ± 0.5	0.9 ± 0.3	0.9 ± 0.4	1.3 ± 0.6	<0.001	1.3 ± 0.5	1.0 ± 0.4	0.8 ± 0.3	<0.001	0.8 ± 0.4	1.0 ± 0.4	1.2 ± 0.5	<0.001
Vitamin B_2, mg	0.8 ± 0.3	0.8 ± 0.4	0.7 ± 0.3	0.9 ± 0.3	<0.001	1.0 ± 0.4	0.7 ± 0.3	0.6 ± 0.2	<0.001	0.8 ± 0.4	0.7 ± 0.3	0.7 ± 0.3	<0.001
Vitamin B_3, mg	14.9 ± 6.2	14.2 ± 5.2	14.0 ± 6.0	17.6 ± 6.5	<0.001	19.5 ± 6.4	14.8 ± 5.4	11.5 ± 4.7	<0.001	14.4 ± 6.5	14.9 ± 6.4	15.3 ± 5.1	<0.001
Vitamin B_6, µg	0.4 ± 0.2	0.3 ± 0.2	0.3 ± 0.2	0.5 ± 0.3	<0.001	0.4 ± 0.3	0.3 ± 0.2	0.3 ± 0.2	<0.001	0.3 ± 0.2	0.3 ± 0.2	0.5 ± 0.3	<0.001
Folic acid, µg	190.7 ± 92.1	180.3 ± 85.2	176.7 ± 86.7	231.3 ± 96.2	<0.001	238.7 ± 102.7	187.2 ± 85.8	158.4 ± 77.4	<0.001	190.9 ± 91.1	185.4 ± 96.4	201.2 ± 84.5	<0.001
Vitamin B_{12}, g	1.5 ± 2.8	2.2 ± 3.6	1.5 ± 3.2	1.2 ± 2.5	<0.001	2.0 ± 3.8	1.6 ± 2.7	1.1 ± 2.0	<0.001	2.4 ± 2.8	1.5 ± 2.5	0.7 ± 1.8	<0.001
Vitamin C, mg	82.0 ± 68.1	66.3 ± 46.6	77.2 ± 55.2	104.4 ± 102.6	<0.001	95.7 ± 72.6	80.0 ± 76.4	74.6 ± 50.5	<0.001	84.1 ± 61.6	81.7 ± 88.8	83.7 ± 56.5	<0.001
Vitamin E, mg	30.9 ± 23.0	21.3 ± 15.5	27.2 ± 19.1	46.5 ± 29.2	<0.001	31.8 ± 25.9	31.0 ± 23.7	29.8 ± 18.1	<0.001	31.9 ± 20.0	31.6 ± 26.6	28.1 ± 21.1	<0.001
Na, mg	5645.1 ± 16455.1	4774.7 ± 6097.7	5441.5 ± 13849.1	6955.8 ± 29110.1	<0.001	7664.5 ± 33378.0	5214.0 ± 8646.8	4775.5 ± 4276.7	<0.001	5039.3 ± 4582.4	5415.8 ± 5676.9	6583.1 ± 31518.5	<0.001

K, mg	1658.7 ± 850.4	1424.4 ± 520.4	1515.7 ± 646.3	2194.0 ± 1255.3	<0.001	2037.2 ± 785.4	1648.5 ± 969.6	1385.2 ± 569.5	<0.001	1675.4 ± 794.9	1630.5 ± 1124.4	1732.7 ± 672.5	<0.001
Mg, mg	312.2 ± 138.8	248.1 ± 78.6	284.5 ± 103.5	433.7 ± 177.8	<0.001	389.7 ± 133.7	305.7 ± 144.3	263.0 ± 103.3	<0.001	272.4 ± 114.6	305.5 ± 147.9	362.2 ± 135.9	<0.001
Fe, mg	22.6 ± 11.7	19.2 ± 8.9	21.0 ± 9.2	29.7 ± 13.6	<0.001	29.7 ± 14.7	22.0 ± 11.2	18.0 ± 7.6	<0.001	20.7 ± 14.2	22.5 ± 11.3	24.8 ± 10.7	<0.001
Zn, mg	11.5 ± 4.3	10.6 ± 3.3	10.7 ± 4.0	14.1 ± 4.9	<0.001	15.0 ± 4.3	11.4 ± 3.9	9.0 ± 3.0	<0.001	10.3 ± 4.0	11.6 ± 4.6	12.5 ± 3.8	<0.001
Se, μg	41.3 ± 26.0	45.3 ± 26.8	38.0 ± 21.6	45.5 ± 26.6	<0.001	55.2 ± 37.0	42.0 ± 23.3	30.3 ± 15.2	<0.001	45.3 ± 21.8	41.6 ± 27.8	38.2 ± 30.0	<0.001

[a] Continuous variables were presented as mean ± SD. [b] Kruskal-Wallis rank-sum analysis was used to test significant differences across different quintiles of plant-based diet indices. Abbreviations: E, energy; Fe, iron; hPDI, healthy PDI; K, potassium; Mg, magnesium; MUFA, monounsaturated fatty acid; Na, sodium; PDI, plant-based diet index; PUFA, polyunsaturated fatty acid; Q, quintiles; RE, retinol equivalent; SD, standard deviation; Se, selenium; SFA, saturated fatty acid; uPDI, unhealthy PDI; Zn, zinc.

Table S5. Baseline daily intakes of nutrients and food groups of 15,318 Chinese adults in cohort B who participated in the China Health and Nutrition Survey 1997-2015 wave based on quintiles of plant-based diet indices [a].

Variables	All	Quintiles of PDI			P^{b}	Quintiles of hPDI			P^{b}	Quintiles of uPDI			P^{b}
		Q1	Q3	Q5		Q1	Q3	Q5		Q1	Q3	Q5	
Total energy, kcal	2213.0 ± 741.5	1802.0 ± 478.6	2064.5 ± 663.4	2809.0 ± 713.8	<0.001	3004.6 ± 681.9	2219.2 ± 564.6	1566.4 ± 400.8	<0.001	2022.7 ± 699.4	2259.4 ± 746.8	2322.7 ± 725.0	<0.001
Carbohydrate, % E	56.0 ± 13.4	51.0 ± 12.1	56.0 ± 13.5	60.4 ± 12.7	<0.001	58.0 ± 13.2	56.2 ± 13.2	54.3 ± 13.4	<0.001	43.7 ± 9.7	53.9 ± 10.5	69.5 ± 9.1	<0.001
Protein, % E	12.3 ± 3.0	13.5 ± 3.4	12.3 ± 2.9	11.4 ± 2.4	<0.001	11.8 ± 2.9	12.4 ± 3.0	12.5 ± 3.0	<0.001	14.4 ± 3.3	12.2 ± 2.8	10.9 ± 2.1	<0.001
Fat, % E	30.0 ± 12.8	33.8 ± 11.9	29.9 ± 13.0	26.4 ± 12.4	<0.001	27.9 ± 12.6	29.8 ± 12.6	31.7 ± 13.0	<0.001	39.5 ± 10.1	32.2 ± 11.1	18.4 ± 9.6	<0.001
SFA, % E	7.0 ± 3.6	9.1 ± 3.9	7.0 ± 3.4	5.4 ± 2.9	<0.001	7.6 ± 4.2	6.9 ± 3.5	6.7 ± 3.2	<0.001	9.3 ± 2.8	7.7 ± 3.4	4.1 ± 2.6	<0.001
MUFA, % E	11.9 ± 6.2	14.1 ± 5.8	12.0 ± 6.3	9.7 ± 5.8	<0.001	11.7 ± 6.1	11.8 ± 6.1	12.0 ± 6.4	0.22	15.6 ± 5.5	13.1 ± 5.7	6.7 ± 4.3	<0.001
PUFA, % E	7.6 ± 4.8	6.8 ± 4.3	7.5 ± 4.8	8.1 ± 5.0	<0.001	5.4 ± 3.8	7.6 ± 4.6	9.4 ± 5.0	<0.001	10.3 ± 4.6	7.7 ± 4.7	5.1 ± 4.1	<0.001
Cholesterol, mg	157.7 ± 180.2	228.3 ± 201.6	155.1 ± 181.0	108.7 ± 156.2	<0.001	236.1 ± 238.8	157.4 ± 172.1	91.0 ± 116.2	<0.001	229.0 ± 194.5	190.3 ± 198.5	58.2 ± 105.2	<0.001
Dietary fiber, g	11.7 ± 8.9	7.7 ± 5.4	10.7 ± 7.9	17.0 ± 10.6	<0.001	13.8 ± 11.4	12.0 ± 9.0	10.0 ± 5.9	<0.001	12.0 ± 8.5	11.2 ± 8.7	12.8 ± 9.7	<0.001
Vitamin A, RE	472.0 ± 786.6	551.6 ± 1255.9	472.1 ± 653.9	412.1 ± 605.3	<0.001	605.7 ± 1381.1	460.9 ± 503.8	386.1 ± 549.6	<0.001	645.1 ± 701.6	511.7 ± 1183.4	273.1 ± 321.0	<0.001
Vitamin B_1, mg	1.0 ± 0.5	0.8 ± 0.3	0.9 ± 0.4	1.3 ± 0.6	<0.001	1.3 ± 0.5	1.0 ± 0.5	0.7 ± 0.3	<0.001	0.9 ± 0.4	1.0 ± 0.4	1.1 ± 0.5	<0.001
Vitamin B_2, mg	0.8 ± 0.3	0.7 ± 0.4	0.7 ± 0.3	0.8 ± 0.3	<0.001	0.9 ± 0.4	0.8 ± 0.3	0.6 ± 0.2	<0.001	0.9 ± 0.4	0.8 ± 0.4	0.6 ± 0.3	<0.001
Vitamin B_3, mg	14.8 ± 6.2	13.3 ± 5.3	14.0 ± 6.2	17.5 ± 6.6	<0.001	19.3 ± 6.4	15.0 ± 5.4	10.7 ± 4.6	<0.001	15.6 ± 7.0	15.1 ± 6.5	14.1 ± 5.2	<0.001
Vitamin B_6, µg	0.4 ± 0.2	0.2 ± 0.2	0.3 ± 0.2	0.5 ± 0.3	<0.001	0.4 ± 0.3	0.4 ± 0.2	0.3 ± 0.2	<0.001	0.3 ± 0.2	0.3 ± 0.2	0.4 ± 0.3	<0.001
Folic acid, µg	190.8 ± 92.5	170.8 ± 88.1	178.1 ± 84.1	229.4 ± 96.0	<0.001	237.7 ± 102.3	193.4 ± 90.1	151.2 ± 78.5	<0.001	205.3 ± 98.1	190.2 ± 94.0	185.0 ± 85.8	<0.001
Vitamin B_{12}, g	1.6 ± 2.8	2.0 ± 3.3	1.6 ± 2.8	1.2 ± 2.5	<0.001	2.0 ± 3.8	1.5 ± 2.6	1.2 ± 2.0	<0.001	2.6 ± 3.0	1.7 ± 3.2	0.6 ± 1.6	<0.001
Vitamin C, mg	82.2 ± 69.3	63.1 ± 45.6	77.1 ± 53.6	104.8 ± 98.4	<0.001	94.2 ± 73.8	84.2 ± 80.4	71.3 ± 51.5	<0.001	90.5 ± 76.8	81.9 ± 89.4	78.8 ± 55.2	<0.001

Vitamin E, mg	31.0 ± 23.0	20.4 ± 15.1	27.6 ± 18.9	45.1 ± 28.5	<0.001	30.8 ± 25.2	32.9 ± 24.6	27.7 ± 16.5	<0.001	36.1 ± 22.4	32.7 ± 26.0	24.9 ± 19.2	<0.001
Na, mg	5618.5 ± 16111.0	4742.5 ± 12357.6	5324.0 ± 11153.7	6706.3 ± 26949.3	<0.001	7635.9 ± 33267.3	5490.7 ± 12726.0	4494.8 ± 3851.4	<0.001	5408.4 ± 4648.4	5603.6 ± 8299.1	5397.7 ± 15649.3	<0.001
K, mg	1662.8 ± 872.4	1339.0 ± 526.6	1530.4 ± 662.6	2151.1 ± 1212.4	<0.001	2020.0 ± 827.1	1712.4 ± 1037.3	1314.4 ± 558.7	<0.001	1822.5 ± 924.9	1660.6 ± 1137.0	1590.4 ± 641.2	<0.001
Mg, mg	311.7 ± 140.4	233.3 ± 80.7	284.2 ± 107.4	424.4 ± 174.0	<0.001	384.2 ± 133.3	318.6 ± 151.6	245.8 ± 98.1	<0.001	298.6 ± 130.9	310.0 ± 158.1	333.9 ± 131.1	<0.001
Fe, mg	22.6 ± 11.9	17.9 ± 7.9	21.2 ± 9.9	29.0 ± 13.2	<0.001	29.2 ± 12.4	23.0 ± 12.0	17.0 ± 7.7	<0.001	22.7 ± 14.8	22.9 ± 11.9	22.6 ± 10.3	0.025
Zn, mg	11.5 ± 4.4	9.9 ± 3.4	10.8 ± 4.0	14.0 ± 4.9	<0.001	14.8 ± 4.3	11.7 ± 4.0	8.3 ± 2.8	<0.001	11.2 ± 4.3	11.7 ± 4.6	11.4 ± 4.1	<0.001
Se, μg	41.5 ± 25.9	41.8 ± 22.9	39.3 ± 25.1	44.9 ± 27.8	<0.001	55.5 ± 36.7	41.8 ± 22.5	29.1 ± 13.9	<0.001	49.0 ± 23.6	42.0 ± 22.9	35.2 ± 30.2	<0.001
Grains, g	414.0 ± 175.4	421.6 ± 155.0	458.6 ± 195.3	339.9 ± 132.7	<0.001	375.3 ± 132.0	431.4 ± 171.3	415.3 ± 197.7	<0.001	377.1 ± 165.9	418.7 ± 177.3	432.3 ± 170.9	<0.001
Whole grains, g	19.9 ± 58.3	4.2 ± 22.1	14.3 ± 38.1	46.6 ± 97.4	<0.001	8.3 ± 34.7	23.8 ± 73.5	26.5 ± 52.6	<0.001	20.3 ± 47.6	20.1 ± 59.0	19.0 ± 56.4	<0.001
Fruits, g	27.3 ± 72.2	18.5 ± 58.8	27.1 ± 71.8	32.6 ± 84.6	<0.001	15.6 ± 53.8	26.7 ± 81.4	40.5 ± 80.2	<0.001	69.4 ± 95.2	22.7 ± 70.7	4.7 ± 26.9	<0.001
Vegetables, g	272.7 ± 149.9	211.8 ± 113.9	261.4 ± 141.2	335.8 ± 170.1	<0.001	285.9 ± 156.7	275.4 ± 157.0	258.1 ± 140.3	<0.001	297.1 ± 143.1	273.2 ± 144.1	244.2 ± 156.6	<0.001
Nuts, g	3.2 ± 12.4	1.6 ± 8.2	2.7 ± 10.7	5.7 ± 17.7	<0.001	1.6 ± 11.0	3.9 ± 13.1	3.7 ± 11.1	<0.001	8.1 ± 17.9	2.6 ± 10.7	0.5 ± 5.9	<0.001
Legumes, g	49.3 ± 67.5	25.3 ± 41.9	47.0 ± 61.1	73.9 ± 87.7	<0.001	44.3 ± 64.9	50.2 ± 69.2	53.4 ± 66.6	<0.001	70.1 ± 72.8	50.0 ± 67.8	26.4 ± 51.8	<0.001
Vegetable oils, g	32.1 ± 29.1	21.0 ± 20.1	29.0 ± 25.0	44.4 ± 34.4	<0.001	27.8 ± 32.8	34.9 ± 29.7	29.5 ± 19.6	<0.001	38.9 ± 27.1	34.9 ± 33.5	22.7 ± 24.1	<0.001
Tea and coffee, g	1.3 ± 24.8	0.3 ± 10.9	1.2 ± 22.2	2.0 ± 32.5	<0.001	0.4 ± 7.7	1.1 ± 22.3	2.3 ± 33.6	<0.001	5.1 ± 51.8	0.5 ± 13.2	0 ± 0	<0.001
Fruit juices, g	0.2 ± 5.5	0.2 ± 6.4	0.1 ± 3.8	0.1 ± 2.4	0.54	0.1 ± 1.9	0.1 ± 2.9	0 ± 0	0.06	0.3 ± 6.5	0.1 ± 2.6	0.1 ± 5.9	0.11
Refined grains, g	403.1 ± 160.2	308.2 ± 104.3	380.2 ± 127.0	517.1 ± 188.3	<0.001	548.6 ± 180.5	399.5 ± 123.8	290.6 ± 108.4	<0.001	294.7 ± 100.8	388.7 ± 130.2	518.0 ± 181.5	<0.001

Potatoes and starch, g	33.1 ± 62.7	10.6 ± 26.2	25.3 ± 47.5	66.5 ± 91.2	<0.001	56.3 ± 89.9	31.6 ± 58.3	18.3 ± 42.8	<0.001	14.5 ± 27.2	22.8 ± 43.8	70.4 ± 97.7	<0.001
Sugar-sweetened beverages, g	1.6 ± 19.1	0.3 ± 6.1	2.1 ± 26.3	2.5 ± 22.2	<0.001	2.6 ± 27.6	1.3 ± 13.1	0.5 ± 7.1	<0.001	2.2 ± 23.9	1.8 ± 20.3	0.5 ± 9.1	<0.001
Sweets and desserts, g	0.8 ± 7.1	0.3 ± 3.5	0.6 ± 5.5	1.6 ± 10.2	<0.001	1.4 ± 11.4	0.8 ± 6.4	0.4 ± 4.1	<0.001	0.9 ± 6.2	0.7 ± 6.3	0.5 ± 5.2	<0.001
Animal fat, g	6.6 ± 18.5	10.2 ± 20.1	7.0 ± 21.1	3.8 ± 11.8	<0.001	20.6 ± 34.0	4.0 ± 11.3	1.2 ± 5.4	<0.001	4.8 ± 15.6	8.5 ± 21.7	5.0 ± 13.0	<0.001
Dairy, g	16.0 ± 56.1	30.6 ± 74.1	13.4 ± 51.2	9.1 ± 45.5	<0.001	12.0 ± 52.4	18.9 ± 59.5	13.6 ± 51.2	<0.001	56.5 ± 94.0	8.5 ± 43.0	1.3 ± 16.5	<0.001
Eggs, g	23.6 ± 31.9	32.0 ± 33.5	23.1 ± 32.8	17.8 ± 28.9	<0.001	29.1 ± 36.4	24.1 ± 31.1	18.0 ± 26.7	<0.001	38.9 ± 33.3	26.5 ± 35.7	9.5 ± 23.3	<0.001
Fish or seafood, g	19.3 ± 34.2	32.3 ± 37.4	17.4 ± 30.7	10.8 ± 30.3	<0.001	25.6 ± 41.4	19.9 ± 32.1	11.2 ± 25.9	<0.001	39.0 ± 43.0	18.8 ± 33.5	4.1 ± 15.1	<0.001
Meat, g	101.0 ± 125.1	124.5 ± 111.3	102.0 ± 124.8	75.0 ± 116.9	<0.001	156.7 ± 181.6	98.2 ± 104.7	58.2 ± 70.8	<0.001	146.1 ± 149.0	117.7 ± 140.9	40.1 ± 60.9	<0.001

[a] Continuous variables were presented as mean ± SD. [b] Kruskal-Wallis rank-sum analysis was used to test significant differences across different quintiles of plant-based diet indices. Abbreviations: E, energy; Fe, iron; hPDI, healthy PDI; K, potassium; Mg, magnesium; MUFA, monounsaturated fatty acid; Na, sodium; PDI, plant-based diet index; PUFA, polyunsaturated fatty acid; Q, quintiles; SD, standard deviation; RE, retinol equivalent; Se, selenium; SFA, saturated fatty acid; uPDI, unhealthy PDI; Zn, zinc.

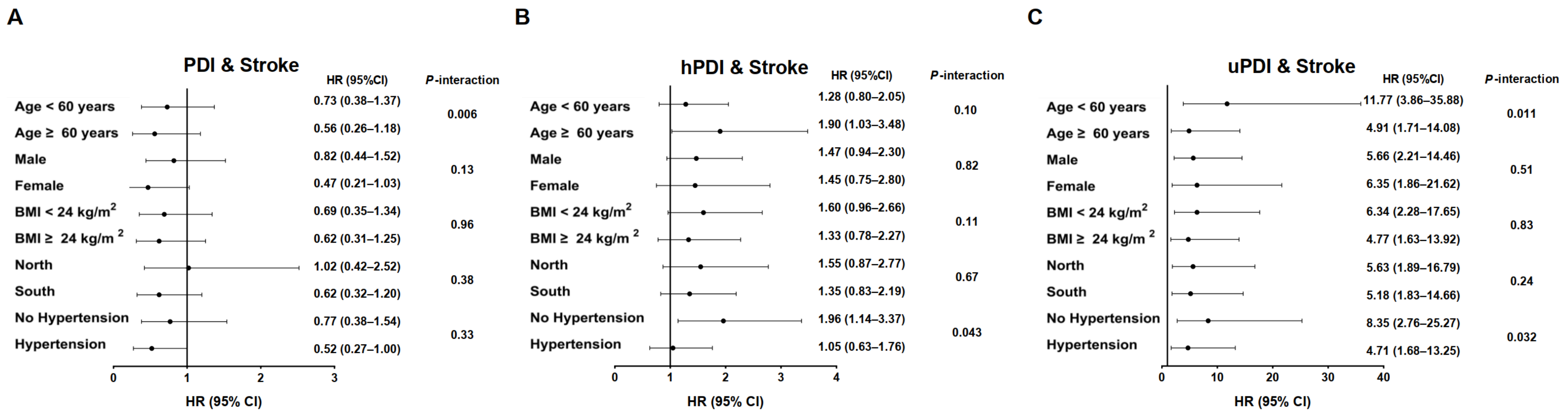

Figure S2. Associations between plant-based diet indices and risk of MI (N = 14,652) in Chinese adults who participated in the China Health and Nutrition Survey 1997-2015 wave, stratified by age, sex, BMI, region and baseline hypertension history (A: PDI and risk of MI, B: hPDI and risk of MI, C: uPDI and risk of MI). Data were presented as HR (95% CI) of Q5 VS Q1 estimated by Cox proportional hazard regression models. Models adjusted for sex, age, BMI, region, urbanization index, educational level, physical activity, baseline hypertension, smoking status, alcohol intake and total energy intake. Models for stroke also adjusted for the sodium: potassium ratio. Stratification variables were not adjusted as confounding factors in the corresponding models. Abbreviations: BMI, body mass index; CI, confidence interval; hPDI, healthy PDI; HR, hazard ratio; MI, myocardial infarction; PDI, plant-based diet index; Q, quintiles; uPDI, unhealthy PDI. The corresponding numerical data were listed in Table S6.

Table S6. Associations between healthy plant-based diet index and risk of MI, T2D, stroke (N = 14,652) and all-cause mortality (N = 15,318) in Chinese adults who participated in the China Health and Nutrition Survey 1997-2015 wave, stratified by age, sex, BMI, region and baseline hypertension history [a].

Variables	Quartiles					*P*-trend	*P*-interaction
	Q1	Q2	Q3	Q4	Q5		
PDI							
MI							
Age < 60 years	1.00 (Ref)	0.75 (0.46, 1.23)	0.94 (0.58, 1.52)	0.73 (0.41, 1.30)	0.61 (0.30, 1.24)	0.25	0.47
Age ≥ 60 years	1.00 (Ref)	0.72 (0.33, 1.57)	0.71 (0.34, 1.50)	0.65 (0.28, 1.54)	0.35 (0.13, 0.92)	0.043	
Male	1.00 (Ref)	0.57 (0.33, 0.99)	0.99 (0.59, 1.66)	0.48 (0.23, 0.98)	0.33 (0.13, 0.82)	0.08	0.10
Female	1.00 (Ref)	1.00 (0.53, 1.90)	0.69 (0.36, 1.33)	0.86 (0.44, 1.71)	0.61 (0.28, 1.33)	0.20	
BMI < 24 kg/m^2	1.00 (Ref)	0.46 (0.34, 1.22)	1.13 (0.63, 2.03)	0.88 (0.44, 1.78)	0.58 (0.25, 1.35)	0.51	0.14
BMI ≥ 24 kg/m^2	1.00 (Ref)	0.81 (0.47, 1.39)	0.63 (0.36, 1.10)	0.59 (0.31, 1.13)	0.46 (0.22, 0.99)	0.038	
North	1.00 (Ref)	1.04 (0.48, 2.26)	0.97 (0.46, 2.04)	0.82 (0.37, 1.82)	0.55 (0.23, 1.33)	0.09	0.75
South	1.00 (Ref)	0.59 (0.35, 1.00)	0.83 (0.49, 1.40)	0.64 (0.32, 1.29)	0.46 (0.19, 1.11)	0.14	
No Hypertension	1.00 (Ref)	0.71 (0.41, 1.23)	1.14 (0.68, 1.90)	0.81 (0.43, 1.52)	0.62 (0.29, 1.33)	0.46	0.40
Hypertension	1.00 (Ref)	0.71 (0.37, 1.35)	0.52 (0.27, 0.99)	0.48 (0.23, 1.00)	0.33 (0.14, 0.76)	0.009	
T2D							
Age < 60 years	1.00 (Ref)	0.69 (0.55, 0.85)	0.56 (0.44, 0.71)	0.45 (0.34, 0.60)	0.35 (0.25, 0.49)	<0.001	0.66
Age ≥ 60 years	1.00 (Ref)	0.93 (0.57, 1.50)	0.70 (0.43, 1.15)	0.56 (0.32, 0.97)	0.38 (0.21, 0.70)	0.005	
Male	1.00 (Ref)	0.67 (0.52, 0.86)	0.53 (0.41, 0.70)	0.41 (0.29, 0.59)	0.27 (0.17, 0.42)	<0.001	0.91
Female	1.00 (Ref)	0.76 (0.55, 1.05)	0.61 (0.44, 0.84)	0.51 (0.36, 0.73)	0.41 (0.27, 0.61)	<0.001	
BMI < 24 kg/m^2	1.00 (Ref)	0.54 (0.40, 0.74)	0.55 (0.40, 0.76)	0.42 (0.29, 0.61)	0.26 (0.16, 0.41)	<0.001	0.045
BMI ≥ 24 kg/m^2	1.00 (Ref)	0.87 (0.67, 1.11)	0.58 (0.44, 0.77)	0.49 (0.35, 0.67)	0.43 (0.30, 0.63)	<0.001	
North	1.00 (Ref)	0.97 (0.68, 1.40)	0.71 (0.50, 1.02)	0.54 (0.37, 0.80)	0.40 (0.26, 0.61)	<0.001	0.15
South	1.00 (Ref)	0.60 (0.47, 0.77)	0.49 (0.37, 0.64)	0.41 (0.29, 0.59)	0.34 (0.22, 0.53)	<0.001	
No Hypertension	1.00 (Ref)	0.70 (0.55, 0.88)	0.59 (0.46, 0.76)	0.45 (0.33, 0.61)	0.35 (0.24, 0.51)	<0.001	0.83
Hypertension	1.00 (Ref)	0.75 (0.53, 1.07)	0.52 (0.36, 0.75)	0.46 (0.30, 0.70)	0.33 (0.20, 0.53)	<0.001	
Stroke							
Age < 60 years	1.00 (Ref)	1.04 (0.68, 1.59)	0.82 (0.51, 1.29)	1.03 (0.62, 1.73)	0.73 (0.38, 1.37)	0.41	0.006
Age ≥ 60 years	1.00 (Ref)	0.79 (0.43, 1.45)	1.15 (0.66, 2.03)	0.63 (0.32, 1.25)	0.56 (0.26, 1.18)	0.15	
Male	1.00 (Ref)	0.91 (0.61, 1.37)	1.04 (0.69, 1.56)	0.73 (0.43, 1.22)	0.82 (0.44, 1.52)	0.49	0.13
Female	1.00 (Ref)	0.90 (0.46, 1.79)	0.69 (0.35, 1.35)	0.73 (0.36, 1.49)	0.47 (0.21, 1.03)	0.042	
BMI < 24 kg/m^2	1.00 (Ref)	0.88 (0.56, 1.38)	0.90 (0.57, 1.42)	0.75 (0.44, 1.29)	0.69 (0.35, 1.34)	0.16	0.96

Variables	Quartiles					*P*-trend	*P*-interaction
	Q1	Q2	Q3	Q4	Q5		
BMI ≥ 24 kg/m^2	1.00 (Ref)	0.97 (0.55, 1.70)	1.02 (0.59, 1.78)	0.79 (0.42, 1.50)	0.62 (0.31, 1.25)	0.15	
North	1.00 (Ref)	1.79 (0.78, 4.11)	1.80 (0.80, 4.01)	1.27 (0.54, 2.97)	1.02 (0.42, 2.52)	0.26	0.38
South	1.00 (Ref)	0.75 (0.50, 1.14)	0.74 (0.47, 1.16)	0.76 (0.44, 1.31)	0.62 (0.32, 1.20)	0.16	
No Hypertension	1.00 (Ref)	0.96 (0.58, 1.59)	1.02 (0.61, 1.70)	0.89 (0.49, 1.61)	0.77 (0.38, 1.54)	0.51	0.33
Hypertension	1.00 (Ref)	0.83 (0.51, 1.36)	0.84 (0.52, 1.36)	0.65 (0.37, 1.15)	0.52 (0.27, 1.00)	0.05	
All-cause mortality							
Age < 60 years	1.00 (Ref)	1.08 (0.84, 1.39)	0.89 (0.68, 1.16)	0.68 (0.49, 0.95)	0.54 (0.34, 0.85)	0.007	0.31
Age ≥ 60 years	1.00 (Ref)	0.84 (0.62,1.14)	0.85 (0.64, 1.13)	0.79 (0.58,1.07)	0.58 (0.41, 0.82)	0.002	
Male	1.00 (Ref)	0.87 (0.69, 1.10)	0.76 (0.60, 0.96)	0.68 (0.51, 0.91)	0.53 (0.36, 0.76)	<0.001	0.24
Female	1.00 (Ref)	1.05 (0.73, 1.49)	1.00 (0.71, 1.41)	0.83 (0.58, 1.20)	0.65 (0.43, 0.98)	0.012	
BMI < 24 kg/m^2	1.00 (Ref)	0.98 (0.79, 1.23)	0.94 (0.76, 1.18)	0.83 (0.64, 1.07)	0.61 (0.45, 0.84)	0.002	0.53
BMI ≥ 24 kg/m^2	1.00 (Ref)	0.73 (0.49, 1.08)	0.59 (0.40, 0.87)	0.48 (0.31, 0.75)	0.46 (0.27, 0.77)	0.002	
North	1.00 (Ref)	1.08 (0.68, 1.69)	0.74 (0.48, 1.15)	0.48 (0.30, 0.76)	0.33 (0.20, 0.56)	<0.001	0.047
South	1.00 (Ref)	0.87 (0.70, 1.09)	0.89 (0.71, 1.11)	0.91 (0.70, 1.18)	0.74 (0.54, 1.02)	0.09	
No Hypertension	1.00 (Ref)	0.97 (0.77, 1.22)	0.91 (0.72, 1.14)	0.77 (0.58, 1.01)	0.59 (0.42, 0.84)	0.004	0.95
Hypertension	1.00 (Ref)	0.89 (0.63, 1.26)	0.74 (0.53, 1.04)	0.69 (0.48, 1.00)	0.51 (0.33, 0.79)	0.002	
hPDI							
MI							
Age < 60 years	1.00 (Ref)	0.80 (0.47, 1.35)	0.78 (0.47, 1.28)	0.66 (0.39, 1.11)	0.88 (0.53, 1.45)	0.66	0.09
Age ≥ 60 years	1.00 (Ref)	0.62 (0.33, 1.16)	0.51 (0.27, 0.95)	0.84 (0.46, 1.54)	0.37 (0.18, 0.76)	0.034	
Male	1.00 (Ref)	0.57 (0.31, 1.05)	0.75 (0.43, 1.30)	0.74 (0.42, 1.32)	0.72 (0.41, 1.29)	0.55	0.86
Female	1.00 (Ref)	0.83 (0.48, 1.41)	0.64 (0.37, 1.10)	0.65 (0.37, 1.14)	0.59 (0.33, 1.05)	0.06	
BMI < 24 kg/m^2	1.00 (Ref)	0.55 (0.31, 0.97)	0.52 (0.30, 0.91)	0.78 (0.45, 1.33)	0.61 (0.34, 1.08)	0.31	0.33
BMI ≥ 24 kg/m^2	1.00 (Ref)	0.94 (0.54, 1.66)	0.86 (0.50, 1.48)	0.66 (0.37, 1.19)	0.76 (0.43, 1.33)	0.21	
North	1.00 (Ref)	0.43 (0.24, 0.78)	0.51 (0.30, 0.87)	0.62 (0.37, 1.05)	0.49 (0.29, 0.84)	0.11	0.20
South	1.00 (Ref)	1.12 (0.64, 1.97)	0.81 (0.46, 1.44)	0.72 (0.39, 1.34)	0.83(0.45, 1.52)	0.27	
No Hypertension	1.00 (Ref)	0.82 (0.49, 1.37)	0.62 (0.37, 1.04)	0.78 (0.47, 1.30)	0.72 (0.42, 1.23)	0.28	0.74
Hypertension	1.00 (Ref)	0.59 (0.31, 1.12)	0.70 (0.39, 1.26)	0.55 (0.29, 1.06)	0.52 (0.28, 0.97)	0.07	
T2D							
Age < 60 years	1.00 (Ref)	0.92 (0.72, 1.18)	0.79 (0.63, 1.01)	0.78 (0.61, 1.00)	0.86 (0.68, 1.10)	0.18	0.46
Age ≥ 60 years	1.00 (Ref)	0.87 (0.59, 1.29)	0.60 (0.39, 0.91)	0.84 (0.56, 1.26)	0.84 (0.55, 1.30)	0.46	

Variables	Quartiles					P-trend	P-interaction
	Q1	Q2	Q3	Q4	Q5		
Male	1.00 (Ref)	0.89 (0.66, 1.20)	0.72 (0.54, 0.97)	0.74 (0.55, 1.00)	0.74 (0.55, 1.00)	0.039	0.68
Female	1.00 (Ref)	0.88 (0.66, 1.18)	0.74 (0.56, 0.99)	0.78 (0.58, 1.05)	0.87 (0.64, 1.17)	0.33	
BMI < 24 kg/m^2	1.00 (Ref)	0.79 (0.58, 1.08)	0.71 (0.52, 0.97)	0.67 (0.48, 0.93)	0.71 (0.51, 0.98)	0.032	0.61
BMI ≥ 24 kg/m^2	1.00 (Ref)	1.01 (0.77, 1.34)	0.77 (0.58, 1.02)	0.91 (0.69, 1.20)	0.96 (0.73, 1.27)	0.74	
North	1.00 (Ref)	0.81 (0.58, 1.13)	0.84 (0.61, 1.14)	0.84 (0.61, 1.16)	0.98 (0.71, 1.34)	0.65	0.05
South	1.00 (Ref)	0.97 (0.75, 1.27)	0.65 (0.49, 0.86)	0.72 (0.54, 0.96)	0.63 (0.47, 0.86)	<0.001	
No Hypertension	1.00 (Ref)	1.02 (0.79, 1.32)	0.80 (0.62, 1.04)	0.77 (0.59, 1.01)	0.95 (0.73, 1.23)	0.35	0.11
Hypertension	1.00 (Ref)	0.67 (0.47, 0.95)	0.60 (0.43, 0.84)	0.72 (0.51, 1.01)	0.57 (0.40, 0.81)	0.012	
Stroke							
Age < 60 years	1.00 (Ref)	1.08 (0.67, 1.76)	0.95 (0.59, 1.53)	1.12 (0.69, 1.79)	1.28 (0.80, 2.05)	0.24	0.10
Age ≥ 60 years	1.00 (Ref)	1.28 (0.71, 2.33)	1.73 (0.98, 3.07)	1.84 (1.01, 3.36)	1.90 (1.03, 3.48)	0.022	
Male	1.00 (Ref)	1.03 (0.64, 1.65)	1.11 (0.70, 1.75)	1.27 (0.80, 2.01)	1.47(0.94, 2.30)	0.040	0.82
Female	1.00 (Ref)	1.41 (0.75, 2.63)	1.54 (0.84, 2.82)	1.46 (0.78, 2.75)	1.45 (0.75, 2.80)	0.37	
BMI < 24 kg/m^2	1.00 (Ref)	0.98 (0.58, 1.66)	1.48 (0.91, 2.41)	1.27 (0.75, 2.13)	1.60 (0.96, 2.66)	0.039	0.11
BMI ≥ 24 kg/m^2	1.00 (Ref)	1.37 (0.80, 2.34)	0.95 (0.54, 1.64)	1.42 (0.83, 2.42)	1.33 (0.78, 2.27)	0.33	
North	1.00 (Ref)	1.22 (0.67, 2.21)	1.48 (0.84, 2.61)	1.70 (0.96, 3.02)	1.55 (0.87, 2.77)	0.10	0.67
South	1.00 (Ref)	1.15 (0.71, 1.85)	1.04 (0.65, 1.68)	1.03 (0.62, 1.71)	1.35 (0.83, 2.19)	0.30	
No Hypertension	1.00 (Ref)	1.39 (0.80, 2.42)	1.12 (0.64, 1.94)	1.18 (0.67, 2.07)	1.96 (1.14, 3.37)	0.020	0.043
Hypertension	1.00 (Ref)	0.97 (0.58, 1.62)	1.21 (0.74, 1.96)	1.43 (0.87,2.36)	1.05 (0.63, 1.76)	0.58	
All-cause mortality							
Age < 60 years	1.00 (Ref)	0.97 (0.70, 1.33)	1.03 (0.77, 1.38)	1.18 (0.86, 1.61)	1.13 (0.85, 1.52)	0.21	0.91
Age ≥ 60 years	1.00 (Ref)	1.00 (0.80, 1.26)	0.93 (0.74, 1.16)	1.10 (0.86, 1.42)	0.97 (0.77, 1.24)	0.94	
Male	1.00 (Ref)	1.07 (0.83, 1.38)	1.05 (0.82, 1.34)	1.33 (1.02, 1.73)	1.10 (0.85, 1.41)	0.34	0.65
Female	1.00 (Ref)	0.89 (0.68, 1.16)	0.90 (0.70, 1.17)	0.90 (0.67, 1.21)	0.93 (0.71, 1.22)	0.72	
BMI < 24 kg/m^2	1.00 (Ref)	0.85 (0.69, 1.05)	0.95 (0.78, 1.16)	1.00 (0.80, 1.26)	0.98 (0.79, 1.21)	0.68	0.07
BMI ≥ 24 kg/m^2	1.00 (Ref)	1.44 (0.96, 2.17)	1.07 (0.71, 1.62)	1.54 (1.00, 2.35)	1.21 (0.81, 1.82)	0.62	
North	1.00 (Ref)	1.11 (0.78, 1.56)	1.04 (0.75, 1.45)	1.02 (0.71, 1.46)	1.07 (0.77, 1.50)	0.84	0.47
South	1.00 (Ref)	0.89 (0.71, 1.11)	0.94 (0.76, 1.16)	1.15 (0.91, 1.45)	0.98 (0.78, 1.23)	0.62	
No Hypertension	1.00 (Ref)	1.08 (0.85, 1.38)	1.17 (0.93, 1.47)	1.16 (0.90, 1.50)	1.09 (0.86, 1.38)	0.50	0.08
Hypertension	1.00 (Ref)	0.81 (0.61, 1.08)	0.71 (0.53, 0.96)	1.02 (0.75, 1.39)	0.93 (0.70, 1.25)	0.89	
uPDI							

Variables	Quartiles					*P*-trend	*P*-interaction
	Q1	Q2	Q3	Q4	Q5		
MI							
Age < 60 years	1.00 (Ref)	1.59 (0.89, 2.85)	3.02 (1.47, 6.20)	3.88 (1.57, 9.55)	6.70 (2.25, 19.91)	<0.001	0.58
Age ≥ 60 years	1.00 (Ref)	1.65 (0.71, 3.79)	4.01 (1.62, 9.94)	3.85 (1.27, 11.68)	4.99 (1.33, 18.81)	0.013	
Male	1.00 (Ref)	1.63 (0.79, 3.34)	5.08 (2.25, 11.47)	4.83 (1.71, 13.61)	7.57 (2.18, 26.34)	0.001	0.11
Female	1.00 (Ref)	1.62 (0.86, 3.04)	2.14 (0.99, 4.61)	3.16 (1.25, 7.98)	4.64 (1.54, 14.03)	0.005	
BMI < 24 kg/m^2	1.00 (Ref)	3.11 (1.33, 7.30)	6.03 (2.27, 16.05)	6.40 (1.97, 20.82)	10.10 (2.53, 40.33)	0.004	0.25
BMI ≥ 24 kg/m^2	1.00 (Ref)	1.21 (0.66, 2.22)	2.41 (1.18, 4.92)	3.11 (1.28, 7.60)	4.52 (1.53, 13.38)	0.003	
North	1.00 (Ref)	2.32 (1.17, 4.59)	4.07 (1.84, 9.03)	4.31 (1.62, 11.47)	6.67 (2.11, 21.10)	0.002	0.49
South	1.00 (Ref)	1.10 (0.56, 2.17)	2.68 (1.21, 5.94)	3.62 (1.34, 9.80)	5.67 (1.65, 19.45)	0.003	
No Hypertension	1.00 (Ref)	1.53 (0.83, 2.82)	2.88 (1.38, 5.99)	3.07 (1.23, 7.66)	5.76 (1.94, 17.17)	0.002	0.60
Hypertension	1.00 (Ref)	1.77 (0.84, 3.76)	4.11 (1.73, 9.78)	5.22 (1.79, 15.23)	6.07 (1.66, 22.17)	0.003	
T2D							
Age < 60 years	1.00 (Ref)	1.18 (0.94, 1.50)	1.26 (0.98, 1.62)	1.62 (1.27, 2.05)	2.19 (1.70, 2.82)	<0.001	0.012
Age ≥ 60 years	1.00 (Ref)	1.79 (1.17, 2.73)	2.47 (1.61, 3.79)	1.72 (1.09, 2.72)	2.03 (1.26, 3.28)	0.012	
Male	1.00 (Ref)	1.03 (0.77, 1.36)	1.32 (0.99, 1.77)	1.31 (0.97, 1.76)	1.73 (1.27, 2.36)	<0.001	0.30
Female	1.00 (Ref)	1.70 (1.25, 2.31)	1.71 (1.25, 2.35)	2.19 (1.60, 2.99)	2.74 (1.97, 3.82)	<0.001	
BMI < 24 kg/m^2	1.00 (Ref)	1.31 (0.92, 1.87)	1.42 (0.98, 2.05)	1.81 (1.27, 2.57)	2.59 (1.79, 3.74)	<0.001	0.35
BMI ≥ 24 kg/m^2	1.00 (Ref)	1.37 (1.06, 1.76)	1.58 (1.22, 2.05)	1.67 (1.27, 2.18)	1.99 (1.49, 2.65)	<0.001	
North	1.00 (Ref)	1.35 (0.98, 1.86)	1.32 (0.94, 1.85)	1.89 (1.37, 2.59)	2.36 (1.71, 3.26)	<0.001	0.11
South	1.00 (Ref)	1.31 (1.00, 1.72)	1.65 (1.25, 2.17)	1.57 (1.17, 2.10)	1.97 (1.40, 2.76)	<0.001	
No Hypertension	1.00 (Ref)	1.17 (0.91, 1.52)	1.42 (1.10, 1.85)	1.64 (1.27, 2.12)	1.86 (1.40, 2.45)	<0.001	0.19
Hypertension	1.00 (Ref)	1.62 (1.15, 2.27)	1.64 (1.14, 2.37)	1.76 (1.21, 2.56)	2.85 (1.95, 4.17)	<0.001	
Stroke							
Age < 60 years	1.00 (Ref)	2.35 (1.34, 4.13)	4.03 (1.96, 8.31)	5.57 (2.23, 13.89)	11.77 (3.86, 35.88)	<0.001	0.011
Age ≥ 60 years	1.00 (Ref)	2.59 (1.41, 4.74)	3.66 (1.77, 7.54)	5.98 (2.53, 14.15)	4.91(1.71, 14.08)	0.004	
Male	1.00 (Ref)	1.81 (1.11, 2.98)	2.91 (1.58, 5.38)	3.90 (1.80, 8.44)	5.66 (2.21, 14.46)	<0.001	0.51
Female	1.00 (Ref)	3.06 (1.47, 6.38)	3.52 (1.47, 8.42)	5.40 (1.94, 15.00)	6.35 (1.86, 21.62)	0.012	
BMI < 24 kg/m^2	1.00 (Ref)	2.25 (1.25, 4.04)	3.35 (1.67, 6.73)	4.40 (1.88, 10.30)	6.34 (2.28, 17.65)	<0.001	0.83
BMI ≥ 24 kg/m^2	1.00 (Ref)	2.31 (1.21, 3.74)	2.64 (1.30, 5.36)	4.38 (1.83,10.48)	4.77 (1.63, 13.92)	0.003	

Variables	Quartiles					*P*-trend	*P*-interaction
	Q1	Q2	Q3	Q4	Q5		
North	1.00 (Ref)	2.27 (1.19, 4.34)	2.81 (1.32, 6.00)	4.65 (1.89, 11.45)	5.63 (1.89, 16.79)	0.003	0.24
South	1.00 (Ref)	2.07 (1.22, 3.52)	3.25 (1.66, 6.38)	4.02 (1.72, 9.38)	5.18 (1.83, 14.66)	0.002	
No Hypertension	1.00 (Ref)	2.14 (1.18, 3.89)	3.27 (1.55, 6.93)	3.74 (1.47, 9.51)	8.35 (2.76, 25.27)	<0.001	0.032
Hypertension	1.00 (Ref)	2.36 (1.36, 4.12)	3.28 (1.66, 6.46)	5.45 (2.37, 12.52)	4.71 (1.68, 13.25)	0.002	
All-cause mortality							
Age < 60 years	1.00 (Ref)	1.52 (1.07, 2.15)	2.07 (1.37, 3.14)	2.85 (1.75, 4.66)	6.53 (3.66, 11.65)	<0.001	0.67
Age ≥ 60 years	1.00 (Ref)	1.54 (1.09, 2.19)	2.48 (1.69, 3.65)	3.96 (2.56, 6.16)	7.26 (4.36, 12.08)	<0.001	
Male	1.00 (Ref)	1.53 (1.13, 2.08)	2.47 (1.72, 3.54)	3.70 (2.41, 5.67)	7.12 (4.32, 11.74)	<0.001	0.53
Female	1.00 (Ref)	1.53 (1.02, 2.31)	2.27 (1.44, 3.58)	3.15 (1.89, 5.25)	6.57 (3.64, 11.87)	<0.001	
BMI < 24 kg/m^2	1.00 (Ref)	1.44 (1.07, 1.93)	2.07 (1.48, 2.89)	2.98 (2.04, 4.37)	6.27 (4.04, 9.75)	<0.001	0.19
BMI ≥ 24 kg/m^2	1.00 (Ref)	1.67 (1.07, 2.62)	2.85 (1.67, 4.86)	4.04 (2.13, 7.71)	6.03 (2.78, 13.08)	<0.001	
North	1.00 (Ref)	1.73 (1.11, 2.72)	1.99 (1.20, 3.30)	3.15 (1.77, 5.60)	5.86 (3.02, 11.38)	<0.001	0.58
South	1.00 (Ref)	1.43 (1.06, 1.92)	2.55 (1.81, 3.58)	3.59 (2.41, 5.35)	7.42 (4.66, 11.83)	<0.001	
No Hypertension	1.00 (Ref)	1.57 (1.15, 2.14)	2.41 (1.68, 3.47)	3.93 (2.58, 5.97)	8.11 (4.97, 13.24)	<0.001	0.67
Hypertension	1.00 (Ref)	1.38 (0.92, 2.08)	2.25 (1.44, 3.53)	2.64 (1.56, 4.45)	4.99 (2.73, 9.12)	<0.001	

[a] Data were presented as HR (95% CI) estimated by Cox proportional hazard regression models. The data were depicted in Figure 2 and Figure S2. Models adjusted for sex, age, BMI, region, urbanization index, education level, physical activity, baseline hypertension, smoking status, alcohol intake and total energy intake. Models for stroke also adjusted for the sodium: potassium ratio. Stratification variables were not adjusted as confounding factors in the corresponding models. Abbreviations: BMI, body mass index; CI, confidence interval; hPDI, healthy PDI; MI, myocardial infarction; PDI, plant-based diet index; Q, quartiles; Ref, reference; T2D, type 2 diabetes; uPDI, unhealthy PDI.

Table S7. Associations between healthy plant-based diet index and risk of MI, T2D, stroke (N = 14,652) and all-cause mortality (N = 15,318) after positive coding for dairy products and fish and seafood [a].

Variables	Quintiles					*P*-trend
	Q1	Q2	Q3	Q4	Q5	
**MI**						
hPDI (same as in model 2 of Table 3)	1.00 (Ref)	0.71 (0.48 - 1.06)	0.66 (0.45 - 0.97)	0.68 (0.46 - 1.01)	0.63 (0.42 - 0.95)	0.05
hPDI with positive coding for dairy products	1.00 (Ref)	0.68 (0.46 - 1.00)	0.60 (0.39 - 0.91)	0.49 (0.31 - 0.76)	0.53 (0.34 - 0.80)	0.003
hPDI with positive coding for fish and seafood	1.00 (Ref)	0.65 (0.43 - 0.97)	0.79 (0.54 - 1.15)	0.79 (0.53 - 1.17)	0.49 (0.32 - 0.75)	0.009
hPDI with positive coding for dairy products, fish and seafood	1.00 (Ref)	0.95 (0.65 - 1.40)	0.67 (0.43 - 1.03)	0.66 (0.42 - 1.03)	0.62 (0.40 - 0.96)	0.012
**T2D**						
hPDI (same as in model 2 of Table 3)	1.00 (Ref)	0.89 (0.72 - 1.09)	0.73 (0.59 - 0.89)	0.76 (0.62 - 0.94)	0.81 (0.65 - 0.99)	0.039
hPDI with positive coding for dairy products	1.00 (Ref)	0.76 (0.62 - 0.94)	0.77 (0.62 - 0.96)	0.62 (0.49 - 0.78)	0.61 (0.49 - 0.77)	<0.001
hPDI with positive coding for fish and seafood	1.00 (Ref)	0.86 (0.70 - 1.05)	0.70 (0.58 - 0.86)	0.72 (0.59 - 0.89)	0.67 (0.54 - 0.82)	<0.001
hPDI with positive coding for dairy products, fish and seafood	1.00 (Ref)	0.86 (0.70 - 1.05)	0.72 (0.58 - 0.90)	0.62 (0.49 - 0.78)	0.58 (0.46 - 0.72)	<0.001
**Stroke**						
hPDI (same as in model 2 of Table 3)	1.00 (Ref)	1.15 (0.79 - 1.68)	1.22 (0.85 - 1.75)	1.32 (0.91 - 1.92)	1.44 (1.00 - 2.09)	0.038
hPDI with positive coding for dairy products	1.00 (Ref)	1.02 (0.73 - 1.43)	0.90 (0.62 - 1.30)	0.76 (0.51 - 1.13)	1.12 (0.77 - 1.62)	0.75
hPDI with positive coding for fish and seafood	1.00 (Ref)	0.99 (0.69 - 1.41)	1.11 (0.78 - 1.56)	1.31 (0.92 - 1.86)	1.02 (0.71 - 1.47)	0.56
hPDI with positive coding for dairy products, fish and seafood	1.00 (Ref)	1.08 (0.76 - 1.52)	0.82 (0.53 - 1.28)	0.71 (0.41 - 1.24)	0.59 (0.30 - 1.15)	0.08
**All-cause mortality**						
hPDI (same as in model 2 of Table 3)	1.00 (Ref)	0.96 (0.80 - 1.15)	0.97 (0.81 - 1.15)	1.10 (0.90 - 1.33)	1.01 (0.84 - 1.21)	0.61
hPDI with positive coding for dairy products	1.00 (Ref)	0.94 (0.79 - 1.10)	0.83 (0.69 - 1.01)	0.80 (0.66 - 0.97)	0.78 (0.64 - 0.96)	0.008
hPDI with positive coding for fish and seafood	1.00 (Ref)	0.85 (0.72 - 1.01)	0.91 (0.77 - 1.08)	0.84 (0.69 - 1.02)	0.71 (0.59 - 0.85)	<0.001
hPDI with positive coding for dairy products, fish and seafood	1.00 (Ref)	0.87 (0.74 - 1.01)	0.68 (0.57 - 0.82)	0.67 (0.55 - 0.81)	0.54 (0.44 - 0.66)	<0.001

[a] Data were presented as HR (95% CI) estimated by Cox proportional hazard regression models. Models for MI, T2D and all-cause mortality adjusted for sex, age, BMI, region, urbanization index, educational level, physical activity, baseline hypertension, smoking status, alcohol intake and total energy intake. Model for stroke further adjusted for the sodium: potassium ratio. Abbreviations: hPDI, healthy PDI; MI, myocardial infarction; Q, quintiles; Ref, reference; T2D, type 2 diabetes.

Table S8. Associations between revised plant-based diet indices after excluding each food group and risk of MI, T2D, stroke (N = 14,652) and all-cause mortality (N = 15,318) in Chinese adults who participated in the China Health and Nutrition Survey 1997-2015 wave [a].

Variables	MI		T2D		Stroke		All-cause mortality	
	HR (95% CI)	*P*-trend	HR (95% CI)	*P*-trend	HR (95% CI)	*P*-trend	HR (95% CI)	*P*-trend
**PDI**								
**Excluded food group**								
No exclusion	0.48 (0.28 - 0.85)	0.031	0.34 (0.26 - 0.46)	<0.001	0.65 (0.40 - 1.04)	0.08	0.57 (0.44 - 0.74)	<0.001
Healthy plant-based food groups								
Whole grains	0.63 (0.36 - 1.10)	0.15	0.42 (0.32 - 0.56)	<0.001	0.74 (0.46 - 1.19)	0.23	0.65 (0.51 - 0.84)	<0.001
Fruits	0.60 (0.33 - 1.09)	0.23	0.42 (0.31 - 0.57)	<0.001	0.78 (0.48 - 1.28)	0.40	0.66 (0.50 - 0.86)	0.002
Vegetables	0.42 (0.24 - 0.76)	0.019	0.37 (0.27 - 0.50)	<0.001	0.64 (0.39 - 1.05)	0.21	0.59 (0.45 - 0.78)	<0.001
Nuts	0.65 (0.37 - 1.13)	0.49	0.43 (0.32 - 0.57)	<0.001	0.78 (0.49 - 1.25)	0.32	0.62 (0.47 - 0.81)	<0.001
Legumes	0.53 (0.29 - 0.94)	0.10	0.38 (0.28 - 0.51)	<0.001	0.91 (0.57 - 1.48)	0.55	0.72 (0.55 - 0.94)	0.008
Vegetable oil	0.54 (0.30 - 0.96)	0.11	0.37 (0.28 - 0.50)	<0.001	0.59 (0.37 - 0.96)	0.07	0.72 (0.56 - 0.94)	0.019
Tea and coffee	0.45 (0.27 - 0.77)	0.023	0.39 (0.29 - 0.50)	<0.001	0.63 (0.41 - 0.98)	0.09	0.64 (0.50 - 0.82)	<0.001
Unhealthy plant-based food groups								
Fruit juices	0.46 (0.27 - 0.78)	0.022	0.39 (0.30 - 0.50)	<0.001	0.62 (0.40 - 0.95)	0.06	0.58 (0.46 - 0.74)	<0.001
Refined grains	0.52 (0.29 - 0.93)	0.045	0.34 (0.25 - 0.46)	<0.001	0.76 (0.47 - 1.22)	0.25	0.56 (0.43 - 0.74)	<0.001
Potatoes and starch	0.49 (0.27 - 0.90)	0.09	0.50 (0.37 - 0.67)	<0.001	0.82 (0.50 - 1.35)	0.53	0.67 (0.52 - 0.88)	0.003
Sugar-sweetened beverages	0.50 (0.30 - 0.85)	0.044	0.39 (0.29 - 0.51)	<0.001	0.65 (0.43 - 1.01)	0.12	0.68 (0.53 - 0.88)	<0.001
Sweets and desserts	0.49 (0.28 - 0.86)	0.041	0.42 (0.32 - 0.55)	<0.001	0.79 (0.50 - 1.27)	0.46	0.66 (0.51 - 0.85)	<0.001
Animal-based food groups								
Animal fat	0.48 (0.26 - 0.89)	0.018	0.31 (0.23 - 0.42)	<0.001	0.48 (0.29 - 0.79)	0.005	0.56 (0.43 - 0.75)	<0.001
Dairy	0.47 (0.26 - 0.82)	0.021	0.32 (0.24 - 0.43)	<0.001	0.54 (0.34 - 0.88)	0.013	0.48 (0.37 - 0.62)	<0.001
Eggs	0.49 (0.27 - 0.87)	0.032	0.29 (0.22 - 0.39)	<0.001	0.48 (0.30 - 0.78)	0.005	0.42 (0.32 - 0.56)	<0.001
Fish and seafood	0.43 (0.24 - 0.77)	0.006	0.32 (0.24 - 0.43)	<0.001	0.50 (0.31 - 0.81)	0.009	0.41 (0.32 - 0.54)	<0.001
Meat	0.10 (0.04 - 0.25)	<0.001	0.30 (0.22 - 0.41)	<0.001	0.35 (0.21 - 0.58)	<0.001	0.42 (0.32 - 0.55)	<0.001
**hPDI**								
No exclusion	0.63 (0.42 - 0.95)	0.05	0.81 (0.65 - 0.99)	0.039	1.44 (1.00 - 2.09)	0.038	1.01 (0.84 - 1.21)	0.61
Healthy plant-based food groups								
Whole grains	0.71 (0.46 - 1.09)	0.24	0.92 (0.73 - 1.15)	0.48	1.55 (1.05 - 2.27)	0.012	1.17 (0.98 - 1.40)	0.08
Fruits	0.70 (0.47 - 1.05)	0.27	0.94 (0.76 - 1.16)	0.87	1.74 (1.17 - 2.58)	0.002	1.11 (0.92 - 1.34)	0.06
Vegetables	0.78 (0.51 - 1.17)	0.17	0.82 (0.66 - 1.01)	0.07	1.49 (1.02 - 2.18)	0.019	1.11 (0.92 - 1.34)	0.23

Nuts	0.84 (0.54 - 1.29)	0.44	0.84 (0.68 - 1.05)	0.44	1.34 (0.91 - 1.97)	0.032	1.06 (0.88 - 1.27)	0.19
Legumes	0.94 (0.61 - 1.42)	0.51	0.87 (0.71 - 1.08)	0.29	1.40 (0.97 - 2.02)	0.021	1.15 (0.95 - 1.38)	0.07
Vegetable oil	0.69 (0.46 - 1.04)	0.17	0.88 (0.71 - 1.09)	0.28	1.41 (0.98 - 2.05)	0.017	1.32 (1.08 - 1.62)	0.002
Tea and coffee	0.66 (0.42 - 1.02)	0.08	0.79 (0.62 - 1.00)	0.041	1.40 (0.94 - 2.09)	0.09	1.00 (0.82 - 1.22)	0.72
Unhealthy plant-based food groups								
Fruit juices	0.77 (0.52 - 1.12)	0.19	0.89 (0.73 - 1.08)	0.09	1.33 (0.94 - 1.87)	0.044	1.12 (0.92 - 1.36)	0.49
Refined grains	0.71 (0.48 - 1.06)	0.13	0.79 (0.64 - 0.97)	0.025	1.29 (0.91 - 1.82)	0.05	0.96 (0.80 - 1.16)	0.83
Potatoes and starch	0.68 (0.46 - 1.01)	0.09	0.65 (0.52 - 0.80)	<0.001	1.00 (0.51 - 1.98)	0.17	0.86 (0.71 - 1.05)	0.33
Sugar-sweetened beverages	0.73 (0.50 - 1.08)	0.14	0.87 (0.72 - 1.06)	0.038	1.32 (0.93 - 1.87)	0.06	1.01 (0.85 - 1.20)	0.91
Sweets and desserts	0.74 (0.50 - 1.11)	0.15	0.79 (0.65 - 0.96)	0.004	1.19 (0.84 - 1.69)	0.12	0.97 (0.81 - 1.15)	0.69
Animal-based food groups								
Animal fat	0.62 (0.41 - 0.93)	0.015	0.76 (0.62 - 0.94)	0.005	1.34 (0.91 - 1.96)	0.17	0.98 (0.81 - 1.20)	0.97
Dairy	0.66 (0.43 - 1.00)	0.06	0.67 (0.54 - 0.84)	0.001	1.15 (0.79 - 1.67)	0.35	0.89 (0.74 - 1.07)	0.24
Eggs	0.65 (0.43 - 0.98)	0.07	0.77 (0.62 - 0.95)	0.019	1.12 (0.78 - 1.61)	0.33	0.81 (0.67 - 0.97)	0.029
Fish and seafood	0.58 (0.38 - 0.88)	0.032	0.79 (0.64 - 0.97)	0.019	1.27 (0.88 - 1.82)	0.09	0.79 (0.65 - 0.95)	0.11
Meat	0.58 (0.38 - 0.89)	0.023	0.86 (0.69 - 1.07)	0.05	1.23 (0.85 - 1.79)	0.31	0.87 (0.72 - 1.05)	0.15
**uPDI**								
No exclusion	5.90 (2.59 - 13.48)	<0.001	2.18 (1.75 - 2.73)	<0.001	5.96 (2.86 - 12.42)	<0.001	6.87 (4.70 - 10.03)	<0.001
Healthy plant-based food groups								
Whole grains	2.71 (1.69 - 4.34)	<0.001	2.08 (1.63 - 2.66)	<0.001	6.46 (3.01 - 13.85)	<0.001	8.75 (5.82 - 13.15)	<0.001
Fruits	1.76 (1.13 - 2.75)	0.005	1.37 (1.09 - 1.73)	0.010	2.19 (1.45 - 3.30)	<0.001	3.97 (2.72 - 5.81)	<0.001
Vegetables	4.64 (2.07 - 10.42)	<0.001	2.20 (1.75 - 2.77)	<0.001	2.56 (1.74 - 3.77)	<0.001	6.68 (4.55 - 9.79)	<0.001
Nuts	2.11 (1.36 - 3.26)	<0.001	1.76 (1.41 - 2.19)	<0.001	4.70 (2.30 - 9.62)	<0.001	6.95 (4.72 - 10.24)	<0.001
Legumes	4.53 (2.01 - 10.27)	<0.001	1.81 (1.44 - 2.27)	<0.001	2.21 (1.50 - 3.25)	<0.001	7.42 (4.98 - 11.06)	<0.001
Vegetable oil	5.71 (2.52 - 12.91)	<0.001	2.05 (1.64 - 2.58)	<0.001	5.91 (2.85 - 12.27)	<0.001	7.37 (4.96 - 10.96)	<0.001
Tea and coffee	2.79 (1.74 - 4.45)	<0.001	2.23 (1.76 - 2.82)	<0.001	5.03 (2.40 - 10.57)	<0.001	8.12 (5.38 - 12.24)	<0.001
Unhealthy plant-based food groups								
Fruit juices	5.04 (2.33 - 10.88)	<0.001	2.06 (1.66 - 2.57)	<0.001	2.43 (1.69 - 3.49)	<0.001	7.00 (4.77 - 10.27)	<0.001
Refined grains	7.41 (3.17 - 17.31)	<0.001	2.70 (2.14 - 3.42)	<0.001	9.72 (4.52 - 20.90)	<0.001	13.33 (8.64 - 20.56)	<0.001
Potatoes and starch	7.70 (3.38 - 17.53)	<0.001	2.51 (2.00 - 3.15)	<0.001	9.12 (4.40 - 18.91)	<0.001	8.10 (5.49 - 11.95)	<0.001
Sugar-sweetened beverages	5.62 (2.57 - 12.27)	<0.001	2.06 (1.66 - 2.56)	<0.001	2.45 (1.71 - 3.52)	<0.001	8.02 (5.48 - 11.74)	<0.001
Sweets and desserts	5.61 (2.58 - 12.23)	<0.001	2.10 (1.69 - 2.60)	<0.001	6.55 (3.22 - 13.29)	<0.001	8.29 (5.66 - 12.15)	<0.001

Animal-based food groups								
Animal fat	3.01 (1.87 - 4.86)	<0.001	2.17 (1.71 - 2.76)	<0.001	6.00 (2.83 - 12.74)	<0.001	7.99 (5.42 - 11.79)	<0.001
Dairy	4.26 (1.90 - 9.59)	<0.001	1.81 (1.44 - 2.27)	<0.001	5.34 (2.53 - 11.27)	<0.001	7.27 (4.91 - 10.78)	<0.001
Eggs	2.54 (1.61 - 4.01)	<0.001	2.00 (1.58 - 2.53)	<0.001	4.18 (2.06 - 8.49)	<0.001	5.78 (3.89 - 8.59)	<0.001
Fish and seafood	2.30 (1.45 - 3.63)	<0.001	1.80 (1.42 - 2.28)	<0.001	2.07 (1.38 - 3.12)	<0.001	4.67 (3.17 - 6.90)	<0.001
Meat	2.61 (1.68 - 4.04)	<0.001	2.28 (1.82 - 2.86)	<0.001	3.80 (1.87 - 7.74)	<0.001	6.36 (4.24 - 9.54)	<0.001

[a] Data were presented as HR (95% CI) of highest quintile in response to lowest quintile estimated by Cox proportional hazard regression models. Models for MI, T2D and all-cause mortality adjusted for sex, age, BMI, region, urbanization index, educational level, physical activity, baseline hypertension, smoking status, alcohol intake, total energy intake and the excluded food group intake. Model for stroke further adjusted for the sodium: potassium ratio. Abbreviations:hPDI, healthy PDI; MI, myocardial infarction; PDI, plant-based diet index; T2D, type 2 diabetes; uPDI, unhealthy PDI.
